# A COSMIN systematic review of instruments for evaluating health-related quality of life in people with Hereditary Angioedema

**DOI:** 10.1186/s12955-025-02342-6

**Published:** 2025-02-13

**Authors:** Irene Baroni, Giulia Paglione, Giada De Angeli, Miriam Angolani, Edward Callus, Arianna Magon, Gianluca Conte, Stefano Terzoni, Maura Lusignani, Rosario Caruso, Andrea Zanichelli

**Affiliations:** 1 Clinical Research Service, IRCCS Policlinico San Donato, San Donato Milanese, Italy; 2 Clinical Psychology Service, IRCCS Policlinico San Donato, San Donato Milanese, Milan, Italy; 3 Department of Biomedical Sciences for Health, University of Milan, Milan, Italy; 4 Health Professions Research and Development Unit, IRCCS Policlinico San Donato, San Donato Milanese, Italy; 5 Department of Medicine, Angioedema Center, IRCCS Policlinico San Donato, San Donato Milanese, Milan, Italy

**Keywords:** COSMIN, HAE-C1-INH, Health-related quality of life, Hereditary angioedema, Measurement properties, Patient-reported outcome measures, Systematic review

## Abstract

**Background:**

Hereditary angioedema (HAE) adversely affects health-related quality of life (HRQoL). HAE often compromises the HRQoL due to the impact on functional capacity caused by edema, pain, other symptoms, and psychosocial factors. Patient-Reported Outcome Measures (PROMs) focus on HRQoL and are crucial tools for evaluating the burden of the disease and choosing the most appropriate interventions for this population. However, no comprehensive evaluations of the characteristics of the available measurements to assess HRQoL have been conducted for this population.

**Aim:**

To identify, analyze, and summarize the PROMs assessing HRQoL in individuals with HAE-C1-INH, addressing the gap in standardized assessment tools.

**Methods:**

A systematic review was conducted up to December 2023 in PubMed, Scopus, Web of Science, Embase, and CINAHL databases, following PRISMA guidelines without language or time restrictions. Psychometric properties of the identified PROMs were appraised using COSMIN standards, and evidence was synthesized using a modified GRADE approach.

**Results:**

From seven studies, five HRQoL PROMs were identified: two generic (SF-36 and SF-36v2) and three disease-specific (HAE-QoL, HAEA-QoL, and AE-QoL). These PROMs generally lacked comprehensive content, structural and cross-cultural validation, with none meeting the criteria for measurement invariance. This limitation affects their applicability across different demographics and cultures. However, the HAE-QoL and AE-QoL instruments were recognized for having moderate quality evidence, suggesting their potential reliability and validity.

**Conclusions:**

This systematic review provides a moderate recommendation for the use of HAE-QoL and AE-QoL in assessing HRQoL in adults with HAE. Despite identified gaps, the moderate evidence quality for these tools supports their use, pending further validation, involving younger age groups and disease-specific contents in the assessments. Developing culturally and demographically adaptable PROMs is, therefore, a priority to improve the accuracy of PROMs in this field.

**Review registration number:**

PROSPERO registration number is CRD42023440137.

**Supplementary Information:**

The online version contains supplementary material available at 10.1186/s12955-025-02342-6.

## Background

Hereditary Angioedema (HAE) due to C1-inhibitor deficiency (HAE-C1-INH) is a rare and potentially life-threatening disease [1]. The estimated prevalence of HAE is approximately 1 in 50,000 individuals [2]. The primary mediator of HAE symptoms is bradykinin, released following the activation of the contact-Kallikrein system that is not controlled by C1 inhibitor. Activation of the contact system, such as after traumas or stressful situations (triggers), leads to activated Factor XII, which converts prekallikrein to kallikrein. Kallikrein then cleaves high molecular weight kininogen to release bradykinin, the mediator of increased vascular permeability and edema formation [3].

There are two types of HAE-C1-INH. Type 1 HAE, which accounts for approximately 85% of cases, is characterized by low levels of functional and antigenic C1-INH [2]. In type 2 HAE the levels of antigenic C1-INH are normal or elevated, but the protein is dysfunctional [2]. Elevated plasma levels of bradykinin are central to the symptoms of HAE. While sequencing the SERPING1 gene could aid in diagnosis, biochemical testing of C1-INH is effective and less expensive [2, 4–6].

Clinical features of HAE include episodic attacks of skin or mucous tissue swelling, particularly affecting the upper respiratory and gastrointestinal tracts [6]. Abdominal symptoms often include abdominal cramps, vomiting, nausea, diarrhea, and occasionally ascites [6]. Triggers for attacks include trauma, emotional stress, infection, and use of drugs such as ACE-inhibitor and estrogens, although many attacks occur without identifiable triggers [7].

The impact of HAE on health-related quality of life (HRQoL) is profound due to the unpredictability and frequency of attacks, the need for medication, and limitations in normal activities [8, 9]. HRQoL is commonly defined as a dynamic, subjective, and multidimensional construct encompassing physical, psychological, social, and, in some cases, spiritual well-being, as it relates to one’s health or disease status [10]. This multidimensional perspective highlights the need for comprehensive and validated instruments to capture the unique challenges faced by individuals with HAE. In fact, HAE has a significant impact on patients’ functional ability, acknowledging that it causes pain and other disease-related symptoms [8]. More precisely, HAE and its consequences on HRQoL may lead to reduced work productivity in adults and missed opportunities in education and work in younger patients, resulting in increased stress levels [9]. People with HAE also experience higher levels of anxiety and depression and fear the transmission of HAE to their descendants [8, 9, 11].

To accurately assess the HRQoL in people with HAE, Patient-Reported Outcome Measures (PROMs) are essential [8, 12–15]. PROMs could be broadly categorized into generic instruments and condition-specific instruments [16]. Generic instruments, such as the SF-36, are designed to assess HRQoL across a wide range of conditions and populations, enabling comparisons between different diseases or healthy populations [17]. In contrast, condition-specific instruments, such as the HAE-QoL, focus on the unique aspects of HRQoL related to a specific disease, ensuring higher sensitivity and relevance to the target population [17]. However, the available HAE-related HRQoL measures are often considered less comprehensive and robust compared to those for other conditions or healthy people, as they lack thorough content validity, structural validation, cross-cultural adaptability, and consistent responsiveness to clinically important changes [12, 13, 18–20]. In addition, most tools are aimed at an adult population, limiting their applicability to younger populations [12, 13, 18–20].

Thus far, no comprehensive evaluations of the characteristics of the available measurements to assess HRQoL have been conducted. This gap significantly limits the choice of proper assessment tools in both clinical practice and clinical trials. As HRQoL is often included as a secondary outcome in many clinical trials, the lack of validated and reliable PROMs hampers the ability to effectively measure and address the impact of HAE on patients’ HRQoL [21]. A comprehensive evaluation regarding the PROMs for assessing HRQoL could also provide a clear portrait of the availability of these measurements in non-English speaking contexts, focusing on the quality of the measurement properties. Therefore, addressing this gap is crucial to enhance the current knowledge regarding the assessment of HRQoL in patients with HAE. Hence, this review aims to identify, analyze, and summarize the PROMs that assess HRQoL in people with HAE and their measurement proprieties.

## Methods

In this systematic review, the COSMIN methodology for systematic reviews of Patient‐Reported Outcome Measures (PROM_S_) and the Preferred Reporting Items for Systematic Reviews and Meta-Analyses (PRISMA) were used as methodological guides [22–24]. The protocol of this systematic review has been registered in PROSPERO (CRD42023440137). The PRISMA 2020 checklist and the COSMIN definitions of measurement properties followed in this study are reported in Supplementary File 1 and Supplementary File 2, respectively.

### Phase 1: systematic literature search

The inclusion criteria were as follows: (a) article or book chapter/book reporting data about people (any age) with a diagnosis of HAE (any type), (b) article or book chapter/book reporting data on any type of instrument (i.e., questionnaire, inventory) and all PROMs which assess HRQoL; all measurement properties (i.e., structural validity, internal consistency, cross‐cultural validity\measurement invariance, reliability, measurement error, criterion validity, hypotheses testing for construct validity, and responsiveness) were considered eligible. No language or time restrictions were applied. Studies on other constructs or in populations different from that of the present study, as well as studies whose full texts were not accessible or were in the form of unpublished manuscripts, conference proceedings, dissertations, and any other type of secondary source, were excluded.

Five databases (PubMed, Scopus, Web of Science, Embase, and CINAHL) were systematically searched up to December 2023 for published articles. The search strategy was developed and conducted using a combination of keywords, database-specific subject headings, and psychometric properties through the comprehensive and sensitive PROMs filter for the PubMed database validated by the COSMIN group and adapted for the other databases (Supplementary file 3) [25].

**Fig. 1:**
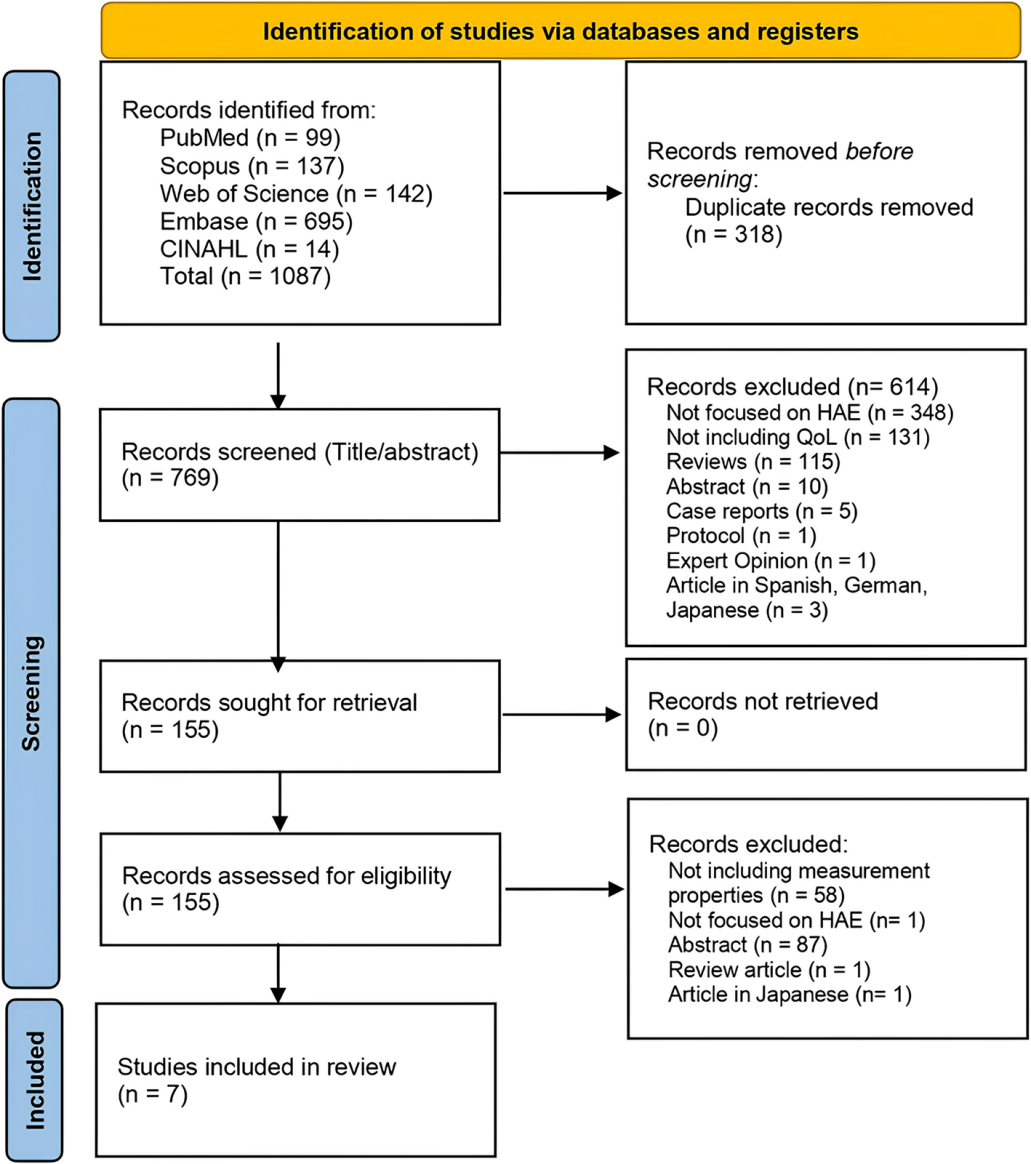
PRISMA 2020 flow diagram

Two authors (IB and GP) independently performed the inclusion selection process by screening titles and abstracts to determine relevant articles for full-text review. A third author and a consensus discussion resolved possible disagreements. There were specific justifications for excluding eligible records. The selection process was represented in a PRISMA 2020 flow diagram (Fig. 1).

The “coding” process was done according to the COSMIN guideline for systematic reviews of PROMs. Specifically, this included: (a) a table on the characteristics of the included PROMs (see Table 1), and (b) a table on the characteristics of the included study populations (Supplementary File 4) [24].

**Table 1:** Characteristics of the included PROMs

PROM*	First Author (year)	(Sub)scale (s) (number of items)	Response options	Scoring; M (SD)	Recall period	Mode of administration	Psychometric proprieties reported
SF-36	Gomide MA et al. (2013) [26]	36 items 8 dimensions: physical functioning (PF), role physical (RP), bodily pain (BP), general health (GH), vitality (VT), social functioning (SF), role emotional (RE) and mental health (MH)	The response scales and the number of response options range from 3-points (PF) to 6-points (VT, MH) Likert scale. Scores for each domain can range from 0 to 100, higher scores indicating better health state	PF = 75.57 (22.61) RP = 60.57 (22.02) BP = 58.10 (30.15) GH = 59.26 (18.71) VT = 51.03 (12.42) SF = 54.29 (13.87) RE = 75.95 (25.14) MH = 65.20 (16.85)	4 weeks	Self-administered	Internal Consistency
SF-36v2	Jindal et al. (2017) [27]	36 items 8 dimensions: physical functioning (PF), role physical (RP), bodily pain (BP), general health (GH), vitality (VT), social functioning (SF), role emotional (RE) and mental health (MH)	The response scales and the number of response options range from 3-points (PF) to 6-points (VT, MH) Likert scale. Scores for each domain can range from 0 to 100, higher scores indicating better health state	PF = 87.86 (16.25) RP = 84.23 (23.19) BP = 67.90 (21.79) GH = 61.05 (23.39) VT = 57.74 (18.32) SF = 85.12 (27.28) RE = 88.89 (24.49) MH = 76.19 (16.95)	4 weeks	Self-administered	Internal Consistency, Floor/Ceiling effects
	Palao‑Ocharan et al. (2022) [28]	36 items 8 dimensions: physical functioning (PF), role physical (RP), bodily pain (BP), general health (GH), vitality (VT), social functioning (SF), role emotional (RE) and mental health (MH)	The response scales and the number of response options range from 3-points (PF) to 6-points (VT, MH) Likert scale. Higher scores indicating better health state	PF = 26.53 (4.15) RP = 15.66 (4.22) BP = 8.01 (3.03) GH = 15.79 (4.86) VT = 12.94 (3.25) SF = 7.91 (2.00) RE = 12.32 (3.01) MH = 18.23 (4.18)	4 weeks	Self-administered	Construct validity (Convergent validity, H-test, Discriminative validity), Internal Consistency, Test–retest reliability, Measurement error (SEM), Minimal clinically important difference (MCID), Floor/Ceiling effects
HAE-QoL	Prior et al. (2012) [29]	44 items 10 dimensions: health conditions, physical functioning, social role, emotional role, physical role, general health, aesthetics, mental health and treatment	5- or 6-point Likert scale depending on the item. Total score ranges from 25–135 (range of scores differs between the various dimensions); higher scores indicate lower impairment	NR	6 months	Self-administered	Face validity, Content validity (PATIENTS),
	Prior et al. (2016) [30]	25 items 7 dimensions: treatment difficulties, physical functioning and health, disease-related stigma, motional role and social functioning, concern about offspring, perceived control over illness, and mental health	5- or 6-point Likert scale depending on the item. Total score ranges from 25–135 (range of scores differs between the various dimensions); higher scores indicate lower impairment	NR	6 months	Self-administered	Structural Validity (EFA), Cross-cultural adaptation, Content validity (EXPERTS), Construct validity (Convergent validity, H-test, Discriminative validity), Criterion validity, Internal consistency, test–retest reliability, Floor/Ceiling effects
HAEA- QoL	Busse et al. (2019) [31]	27 items 2 dimensions: emotional and social well-being “feelings” domain, and an HAE-specific “concerns” domain	4-point Likert scale: 1 = not at all; 2 = a little; 3 = medium; 4 = a lot/severe Higher scores indicated worse outcomes	NR	NR	Self-administered	Face validity, Content validity, Structural validity (EFA), Construct validity (known-groups validity)
AE-QoL	Vanya et al. (2023) [32]	17 items 4 dimensions: functioning, fatigue/mood, fears/shame, and food	5-point Likert scales: never, rarely, sometimes, often, and very often. Total score range from 0 to 100, with higher scores indicating greater impairment	Total = 46.60 (20.89)	4 weeks	Self-administered	Content validity, Construct validity (Convergent validity, H-test, Discriminant validity), Criterion validity, Internal consistency, Test–retest reliability, Measurement error (SEM), Sensitivity to change

Legend: * Each version of a PROM is considered a separate PROM.

*SF-36v2* Short Form 36-item Health Survey Version 2.0, *HAE-QoL* Hereditary angioedema quality of life, *SF-36* Short Form 36-item Health Survey

*HAEA-QoL* Hereditary Angioedema Association quality of life, *AE-QoL* Angioedema Quality of Life Questionnaire, *NR* Not Reported.

Note: Examples of content investigated by items from each PROM: SF-36/SF-36v2: General health perceptions, limitations in physical functioning (e.g., vigorous activities), role limitations due to physical and emotional problems, bodily pain, and social functioning. HAE-QoL: Impact of treatment difficulties, disease-related stigma, physical functioning and health, emotional and social functioning, and concerns about disease transmission to offspring. HAEA-QoL: Frequency of hereditary angioedema attacks, their emotional impact, and the effect on daily life activities and work productivity. AE-QoL: Disease burden related to mental health, social embarrassment, fatigue, and activity restrictions.

### Phase 2: Assessment and quality appraisal of measurement properties

Assessment of each instrument’s measurement properties was done through (a) assessment of the methodological quality of the studies based on the COSMIN Risk of Bias checklist, (b) rating results for single studies using the updated criteria for good measurement properties, (c) summing up the results of all studies for each instrument, and (d) grading the quality of evidence for each measurement property using the modified GRADE approach (Supplementary File 5) [33]. Also, in this phase, all assessments were made by two reviewers independently, and disagreements were resolved through discussion or consulting a third researcher.

The COSMIN Risk of Bias checklist was used to assess the methodological quality of the study’s measurement properties [34]. The COSMIN Risk of Bias Checklist has ten boxes (Supplementary File 6) for assessment. To assess the methodological quality of each study, the measurement properties were specified first, and then relevant boxes were selected. As per guidelines, each measurement standard was scored using a four-point scale consisting of “very good,” “adequate,” “doubtful,” and “inadequate”; the lowest score of each item determined the overall score of each box based on the suggested “worst score counts” principle [34]. Rating of the studies for measurement properties was done separately using the updated criteria for good measurement properties, and the results were rated sufficient ( +), insufficient (-), or indeterminate (?) (see Table 2).

**Table 2:** Results of studies on measurement properties

First Author (year)	Content Validity			Structural validity			Internal consistency			Cross-cultural validity\measurement invariance			Reliability			Measurement error			Criterion validity			Hypotheses testing for construct validity			Responsiveness		
	n	M	Q	n	M	Q	n	M	Q	n	M	Q	n	M	Q	n	M	Q	n	M	Q	n	M	Q	n	M	Q
**SF-36**																											
Gomide MA et al. (2013) [26] Brazilian population	NA	N	NA	NA	N	NA	35	V	Cronb. α = 0.66–0.94 (-)	NA	N	NA	NA	N	NA	NA	N	NA	NA	N	NA	NA	N	NA	NA	N	NA
**SF-36v2**																											
Jindal et al. (2017) [27] Canadian popolation (2 lang.)	NA	N	NA	NA	N	NA	21	V	Cronb. α = 0.83–0.94 ( +)	NA	N	NA	NA	N	NA	NA	N	NA	NA	N	NA	NA	N	NA	NA	N	NA
Palao-Ocharan et al. (2022) [28] International population (11 lang.)	NA	N	NA	NA	N	NA	290	V	Cronb. α = 0.82–0.93 ( +)	NA	N	NA	20	A	ICC = 0.76–0.96 ( +)	290	A	?	290	A	r = 0.45–0.64 (-)	290	A	Results in line with 5 hypo's (5 +) Result not in line with 1 hypo (1‐)	NA	N	NA
**HAE-QoL**																											
Prior et al. (2012) [29] Spanish population	45 + 8	A	+	NA	N	NA	NA	N	NA	NA	N	NA	NA	N	NA	NA	N	NA	NA	N	NA	NA	N	NA	NA	N	NA
Prior et al. (2016) [30] International population (17 lang.)	15	D	-	290	A	[EFA] RMSEA < 0.05; FL > 0.4 ( +)	290	V	Cronb. α = 0.71–0.92 ( +)	290	D	?	37	A	ICC = 0.70–0.90 ( +)	NA	N	NA	290	A	r = 0.45–0.64 (-)	290	A	Results in line with 3 hypo's (3 +)	NA	N	NA
**HAEA-QoL**																											
Busse et al. (2019) [31] US population	NR	D	-	168	A	[EFA] RMSEA = 0.072; FL > 0.4; CFI/TLI > 0.95 ( +)	NA	N	NA	NA	N	NA	NA	N	NA	NA	N	NA	NA	N	NA	NA	N	NA	NA	N	NA
**AE-QoL**																											
Vanya et al. (2023) [32] International population (6 lang.)	40 + 7	D	-	NA	N	NA	64	V	Cronb. α = 0.78–0.92 ( +)	NA	N	NA	64	A	ICC = 0.81–0.89 ( +)	64	A	?	NA	N	NA	64	A	Results in line with 4 hypo's (4 +)	64	A	Results in line with 1 hypo's (1 +)

Legend. *SF-36v2* Short Form 36-item Health Survey Version 2.0, *HAE-QoL* Hereditary angioedema quality of life, *SF-36* Short Form 36-item Health Survey, *HAEA- QoL* Hereditary Angioedema Association quality of life, *AE-QoL* Angioedema Quality of Life Questionnaire, *QOE* Overall Quality of Evidence Grading, *EFA *Exploratory factor analysis, *RMSEA* root mean square error of approximation, *FL* Factor loading, *ICC* Intraclass Correlation Coefficient, *r* Pearson's correlation coefficient, *ρ* Spearman's rank correlation coefficient

The summarized evidence was finally graded using the modified Grading of Recommendations Assessment, Development and Evaluation (GRADE) [33]. This process was aimed to determine the overall quality of the instrument, and evidence quality is graded as high, moderate, low, or very low (see Table 4). Also, two authors (IB and GP) rated and graded each measurement property’s summarised results in this phase.

### Phase 3: Selection of the most suitable self-report tool

Suitable instruments were selected based on the results of the previous steps explained and the assessment of interpretability and feasibility, even if they are not considered measurement properties, as suggested by the COSMIN methodology [33].

## Results

The PRISMA flow diagram is presented in Fig. 1. Of 769 non-duplicated records, 614 were excluded after screening with eligibility criteria (see Supplementary File 3). A total of 155 full-text articles were assessed, resulting in seven studies that met the inclusion criteria [26–32], identifying five HRQoL PROMs. Specifically, two generic measurements were found, which are Short Form 36-item Health Survey (SF-36) and Short Form 36-item Health Survey Version 2.0 (SF-36v2), and three disease-specific: Hereditary angioedema quality of life (HAE-QoL); Hereditary Angioedema Association quality of life (HAEA- QoL); Angioedema Quality of Life Questionnaire (AE-QoL) tools. All articles included in the systematic review were published in English.

The studies included a total of 879 participants, with sample sizes ranging from 20 to 290 across individual studies. The age range of participants varied from 13 to 74 years, with a predominance of female participants (64%–95.2% across studies). Most studies focused on patients with HAE-C1-INH (type I or II). Geographical coverage included countries such as Brazil, Canada, the United States, Spain, and several European nations, with instruments being administered in native languages. For the generic tools, SF-36 was validated in a Brazilian study involving 35 participants with a mean age of 40.7 years, while SF-36v2 was assessed in two studies: a Canadian study with 21 participants and a multicountry study with 290 participants across 12 nations, both involving predominantly female participants. The HAE-QoL was validated in two studies, including a multicenter Spanish study with 45 participants and a broader international study with 290 participants across multiple countries, with mean participant ages of 39 and 41.5 years, respectively. The HAEA-QoL was validated in the United States with 168 participants, where 73.2% were female, and the sample included 7.1% of participants under the age of 18. The AE-QoL was assessed in a two-phase study that involved 40 participants in phase one and 64 participants in phase two, with a mean age of 41 years and an international focus, including Canada, France, Spain, Germany, the United Kingdom, and the United States (see Supplementary File 4).

### Characteristics of the Self-Report Tools

Table 1 displays the description of each tool and the measurement properties reported by the validation articles.

The SF-36 was recently validated in patients with HAE in 2013 [26]. The SF-36v2, a second version of SF-36 was introduced to correct some “deficiencies” in the original version. The original version has been widely used since 2000 and has been psychometrically validated in more than 400 articles [28]. Also, this version has been recently validated in the adult population with HAE [27, 28].

The HAE-QoL, developed in Spain to specifically measure HRQoL in adult people with HAE [29], has been validated in 17 countries [30]. It represents the first disease-specific questionnaire designed for this population and comprises 25 items on seven multi-item dimensions: physical functioning and health, disease-related stigma, emotional role and social functioning, concern about offspring, perceived control over illness, and mental health. In 2019, the HAEA-QoL was specifically developed to assess the impact of HAE on HRQoL in patients from the United States of America (US) [31]. It comprises 27 items on two multi-item dimensions: emotional and social well-being “feelings” and HAE-specific “concern”. Finally, the AE-QoL questionnaire, initially developed to measure HRQoL in patients with bradykinin- and histamine-mediated angioedema, has been recently validated to be also used in adults with HAE in 6 countries [32]. It is a brief self-report tool containing 17 items on four multi-item dimensions: functioning, fatigue/mood, fears/shame, and food. Its main difference with the HAE-specific tools is the gap in assessing HAE’s genetic and mortality risk.

### Quality Appraisal of Measurement Properties

The assessment and quality appraisal are reported in Table 2.

#### Content validity

Studies related to the validation process of the generic tools (SF-36 and SF-36v2) did not report data on content validity, as they neither asked patients nor professionals about the relevance, comprehensiveness, or comprehensibility of the PROM items for HAE [26–28]. Two other studies, focused on the disease-specific measures HAE-QoL and AE-QoL, were rated as having doubtful methodological quality due to unclear descriptions of the methods used to assess relevance, comprehensiveness, and comprehensibility [31, 32]. Both studies assessed relevance by asking patients, but only Vanya et al. asked professionals about relevance [32]. Moreover, patients’ comprehensibility and comprehensiveness, as well as professionals’ comprehensiveness, were assessed only by Vanya et al. 2023. Sufficient content validity evidence ( +) was provided in 1 study [29], which also had adequate or very good methodological quality scores. These findings contrast with the studies related to the generic measures (SF-36 and SF-36v2), which did not report any data on content validity [26–28], as they neither engaged patients nor professionals in evaluating relevance, comprehensiveness, or comprehensibility. While the generic tools omitted these assessments altogether, the disease-specific measures attempted to assess content validity but lacked adequate methodological rigor.

#### Structural validity

All studies related to the generic tools (SF-36 and SF-36v2) validation process provided no evidence of structural validity [26–28]. Also, Vanya et al. have not assessed structural validity [32]. In the remaining two studies, methodological quality scores were adequate or very good [30, 31], with appropriate factor analysis and sample sizes, and rated sufficient evidence quality ( +).

#### Internal Consistency

In most of the studies, methodological quality scores were very good, with sufficient evidence quality ( +), reporting Cronbach’s alpha ≥ 0.70 for each sub-dimension [27, 28, 30, 32]. Gomide et al. presented adequate or very good methodological quality scores but insufficient evidence quality (-) due to a Cronbach’s alpha < 0.70 for some sub-dimensions [26]. Busse et al. did not report data on internal consistency [31].

#### Cross-cultural validity and measurement invariance

Most studies did not report sufficient data on cross-cultural validity and measurement invariance. Only Prior et al. 2016 assessed this property using a cross-cultural adaptation process that involved forward–backward translations and expert consensus meetings [30]. However, the study did not explicitly test measurement invariance, which is a standard statistical approach for evaluating cross-cultural validity. Instead, they relied on qualitative feedback and factor analysis for item evaluation, which did not adequately address measurement invariance across cultural groups. For this reason, the quality of evidence was rated as indeterminate (?) because the cross-cultural validity was not tested using robust measurement invariance approaches, as recommended by the COSMIN guidelines.

#### Reliability

Test–retest reliability was assessed in three studies, with adequate methodological quality and sufficient evidence quality ( +) reporting intraclass correlation (ICC) ≥ 0.70 [28, 30, 32]. The other studies did not assess test–retest reliability.

#### Measurement Error

Most studies did not assess measurement error. Two studies assessed this property with adequate methodological quality but using doubtful analysis approaches [28, 32]. The quality of evidence was then rated indeterminate (?).

#### Criterion validity

Knowing that there is no internationally recognised gold standard tool to measure HRQoL to compare against [18], the HRQoL questionnaires proposed by the author as a comparator were considered the best instruments available for this purpose. So, in this case, two studies had adequate to very good overall methodological quality scores for correlational analysis [28, 30]. However, the quality of evidence was considered insufficient for these studies, which showed a significant correlation with the gold standard but a moderate strength. The other studies did not assess criterion validity.

#### Hypothesis testing for construct validity and Responsiveness

The review team formulated a set of hypotheses for construct validity and responsiveness about expected relationships between the PROM under review and other well‐defined comparators used in the field. Other hypotheses were formulated about expected differences between subgroups, including the expected direction (positive or negative) and magnitude (absolute or relative) of the correlations or differences (see Table 3).

**Table 3:** Hypotheses to evaluate construct validity and responsiveness

1. Correlations with instruments measuring similar constructs should be ≥ 0.40 (positive)
2. Correlations with instruments measuring related, but dissimilar constructs should be lower, i.e. 0.10‐0.40
3. Correlations with instruments measuring unrelated constructs should be < 0.30
4. Correlations of change over time defined under 1, 2, and 3 should be mantained [responsiveness]
5. Meaningful changes between relevant (sub)groups (e.g. patients with expected high vs low levels of HRQoL; pt with LARYNX ATTACKS, or EMERGENCY VISITS, or > N ATTACKS, inadeguate Long-term prophylaxis (LTP) will have significant lower HRQoL)
6. For responsiveness, AUC should be ≥ 0.70

The overall methodological quality score of construct validity was adequate or very good for three studies [28, 30, 32]. Known-groups validity showed that patients with larynx attacks, emergency visits, more frequency of attacks, and inadequate long-term prophylaxis (LTP) had significantly lower HRQoL. Some studies reported positive and significant convergent validity with similar tools, such as SF-36v2 with HAE-QoL and AE-QoL with Sheehan Disability Scale (SDS) [28, 30, 32]. Significant discriminant validity was found with different tools, such as AE-QoL with EQ-5D-5L and EQ-Visual Analog Scale (VAS) [32]. Insufficient evidence quality was only rated for unconfirmed hypotheses [28]. The other studies lacked evidence on hypothesis testing.

Most studies did not report data on responsiveness. Only Vanya et al. assessed this property, with methodological quality scores considered adequate or very good and sufficient evidence quality ( +) adhering to the in-advance formulated hypothesis [32].

### Assessment of interpretability and feasibility

Focusing on interpretability, the main difference between tools is represented by the floor and ceiling effect. Floor and ceiling effects were examined by three studies [27, 28, 30]. SF-36 [27] and SF-36v2 [28] showed a low floor effect (< 5%) in every sub-domain but a very high ceiling effect (> 25%) in most sub-domains. These high-ceiling effects could be related to the low sensitivity of these sub-domains in HAE patients. HAE-QoL showed a low floor effect (< 10%) in every sub-domain but a moderate ceiling effect (from 10 to 25%) in most of the sub-domains [30]. Generally, floor and ceiling effects can indicate insufficient content validity, resulting in insufficient reliability.

The main difference in feasibility between tools seems to be the competition time that is considered “demanding” for the HAE-QoL (about 19 min). Conversely, according to HAE experts, AE-QoL has the shortest time to be completed.

### Recommendation: rating and grading

The overall qualitative rating was performed for all the HRQoL tools using the modified GRADE approach, and the quality of evidence was defined as high (A), moderate (B), low (C), or very low (D). This approach was also used to downgrade evidence when there were concerns about the quality of the evidence (e.g. if the evidence was based on only one inadequate study). The Summary of Findings Table reports the results (see Table 4).

**Table 4:** Summary of Findings

Type of tool (n of studies)	**Content Validity**		**Structural Validity**		**Internal consistency**		**Cross-cultural Validity**		**Reliability**		**Measurement error**		**Hypotheses testing for construct validity**		**Criterion Validity**		**Responsiveness**	
	Rating	QOE	Rating	QOE	Rating	QOE	Rating	QOE	Rating	QOE	Rating	QOE	Rating	QOE	Rating	QOE	Rating	QOE
SF-36	NA	NA	NA	NA	**-**	**Very Low**	NA	NA	NA	NA	NA	NA	NA	NA	NA	NA	NA	NA
(n=1)																		
SF-36v2	NA	NA	NA	NA	**+**	**High**	NA	NA	NA	NA	NA	NA	**+**	**Moderate**	**-**	**Very Low**	NA	NA
(n=2)																		
HAE-QoL	**+/-**	**Moderate**	**+**	**Moderate**	**+**	**Moderate**	**?**	**Low**	**+**	**Moderate**	**?**	**Low**	**+**	**Moderate**	**-**	**Very Low**	NA	NA
(n=2)																		
HAEA-QoL	**-**	**Very low**	**+**	**Moderate**	NA	NA	NA	NA	NA	NA	NA	NA	NA	NA	NA	NA	NA	NA
(n=1)																		
AE-QoL	**-**	**Very low**	NA	NA	**+**	**Moderate**	NA	NA	**+**	**Moderate**	**?**	**Low**	**+**	**Moderate**	NA	NA	**+**	**Moderate**
(n=1)																		

*Legend. SF-36v2 *Short Form 36-item Health Survey Version 2.0, *HAE-QoL* Hereditary angioedema quality of life, *SF-36 *Short Form 36-item Health Survey, *HAEA- QoL *Hereditary Angioedema Association quality of life, *AE-QoL *Angioedema Quality of Life Questionnaire, *QOE *Overall Quality of Evidence Grading

Note. Bold values are the PROMS underwent grading.

Overall rating: + (sufficient); - (insufficient); +/- (inconsistent);? (indeterminate); NA (not applicable)

*Grading QOE*: High. Moderate; Low; Very low according GRADE approach modified

(a) Content Validity [HAE-QoL(n)=2; HAEA-QoL(n)=1; AE-QoL(n)=1]

(b)Structural Validity [HAE-QoL*(n)=1; HAEA-QoL(n)=1]*

(c) Internal Consistency [SF-36*(n)=1; SF-36v2(n)=2; HAE-QoL(n)=1; AE-QoL(n)=1]*

(d) Cross-cultural validity [HAE-QoL(*n*)=1]

(e) Measurement Error [HAE-QoL(n)=1; AE-QoL(n)=1]

(f) Reliability [HAE-QoL(*n*)=1; AE-QoL(*n*)=1]

(g) Hypotheses testing [SF-36v2(n)=1; HAE-QoL(*n*)=1; AE-QoL(*n*)=1]

(h) Criterion Validity [SF-36v2(*n*)=1; HAE-QoL(*n*)=1]

(i) Responsiveness [AE-QoL(*n*)=1]

*SF-36*. This tool had very low-quality evidence for internal consistency. Moreover, only one inadequate study reported this assessment. No other measurement properties were reported. According to COSMIN guidelines, SF-36 has (almost) not been validated for measuring HRQoL in people with HAE. Its performance in all or most relevant quality criteria is unclear, so it is recommended to no longer be used for measuring HRQoL in people with HAE (Grade C).

*SF-36v2*. This tool had high-quality evidence for internal consistency and moderate-quality evidence for hypothesis testing for construct validity. However, it had insufficient ratings and very low quality of evidence for criterion validity. Other measurement properties were not assessed. According to COSMIN guidelines, due to a negative rating and grading in at least one domain and no data on most measurement properties, SF-36v2 is recommended to no longer be used for measuring HRQoL in people with HAE (Grade C).

*HAE-QoL*. This tool had moderate quality evidence for content validity, structural validity, internal consistency, reliability, and hypothesis testing for construct validity with sufficient ratings; low quality of evidence for cross-cultural validity and measurement error with indeterminate ratings; and very low quality of evidence in criterion validity with insufficient ratings. Responsiveness was not assessed. According to COSMIN guidelines, HAE-QoL has a sufficient rating in most of the measurement properties, but performance in content validity is unclear. It has the potential to be recommended in the future, depending on the results of further validation studies (Grade B).

*HAEA-QoL*. This tool had moderate-quality evidence for structural validity with sufficient rating but very low-quality evidence for content validity with insufficient rating. No other measurement properties were reported. According to COSMIN guidelines, HAEA-QoL has (almost) not been validated for measuring HRQoL in people with HAE. Its performance in all or most relevant quality criteria is unclear, so it is not recommended to be used until further validation studies clarify its quality (Grade C).

*AE-QoL*. This tool had moderate quality evidence for internal consistency, reliability, hypothesis testing for construct validity, and responsiveness with sufficient ratings; low-quality evidence for measurement error with indeterminate rating; and very low-quality evidence for content validity with insufficient rating. According to COSMIN guidelines, AE-QoL has a sufficient rating in most of the measurement properties, but performance in content validity is insufficient. It has the potential to be recommended in the future, depending on the results of further validation studies (Grade B).

*Final recommendation*. Considering what is stated in the COSMIN manual [“*only PROMs categorized as “B” are found in this review, the one with the best evidence for content validity could be the one to be provisionally recommended for use until further evidence is provided*”] [23], the authors recommend HAE-QoL for measuring HRQoL in people with HAE.

## Discussion

The current systematic review addresses a key gap in the literature by summarizing the characteristics of tools to assess HRQoL in HAE [2, 8, 9, 11, 13, 15]. HRQoL in HAE has been measured using two generic tools, the SF-36 and SF-36v2, and three disease-specific tools, the HAE-QoL, HAEA-QoL, and AE-QoL. The unpredictability and frequency of HAE attacks, along with their impact on lifestyle and the need for ongoing medication and adjustments, underscore the importance of accurate HRQoL assessment tools [12–14]. Despite this, our review highlights some critical limitations in the available measures. Specifically, the appraisal of the psychometric properties of the available PROMs to measure HRQoL through the COSMIN methodology revealed that the current measurements generally lack comprehensive validation, particularly for cross-cultural contexts [26–32]. Moreover, this review highlights that most tools are focused on adult populations, leaving a significant gap for tools designed for younger patients.

Despite their broad usage, the generic tools SF-36 and SF-36v2 demonstrated significant limitations in their validation for the HAE population [26–28]. The SF-36, in particular, demonstrated very low-quality evidence for internal consistency and lacked a comprehensive assessment of other measurement properties [26]. A key limitation of generic tools, including the SF-36 and SF-36v2, is their inability to comprehensively capture disease-specific impacts, which are essential for understanding the unique HRQoL challenges associated with conditions like HAE. Nevertheless, their generic nature offers distinct advantages, such as enabling comparisons across different diseases and providing valuable inputs for economic evaluations, including cost-utility analyses. While the SF-36v2 showed slightly better performance, both tools demonstrated critical gaps in addressing HAE-specific HRQoL alterations. Based on the current evidence from two studies, further validation and multi-country comparisons are needed to establish their suitability for this purpose. Consequently, the SF-36 and SF-36v2 may not be the most appropriate tools for measuring HRQoL in HAE without further validation and adaptation to this population [27, 28].

In contrast, the disease-specific tools provided more relevant insights but also showed areas needing further validation [29–32]. The HAE-QoL emerged as a potentially reliable tool with moderate quality evidence in several key psychometric properties [29, 30]. However, its content validity remains unclear, and further studies are necessary to confirm its comprehensive applicability. Similarly, AE-QoL showed great promise but had insufficient evidence for content validity, indicating the need for more robust validation efforts [32].

The review’s results underscore the critical need to develop and validate further PROMs specifically tailored to HAE and adaptable across various demographic and cultural contexts. Current tools predominantly focus on adult populations and are primarily validated in English-speaking contexts, leaving significant gaps in HRQoL assessment for younger and non-English-speaking patients. The studies included in this review predominantly focused on adult populations, with age ranges primarily above 18 years and only one study reporting data for participants under 18 years (7.1%). Additionally, while no language restrictions were applied in the inclusion criteria, all included studies were published in English, and most of the tools were validated in English-speaking contexts or translated into a limited number of other languages, such as Spanish, Portuguese, and German (see Supplementary File 4). These gaps underscore the need for further research to develop and validate HRQoL tools specifically designed for younger patients and diverse linguistic and cultural contexts. Addressing these gaps is essential for at least three main reasons.

First, HRQoL could vary significantly across different age groups and cultural backgrounds [35–37]. Younger patients may have different challenges and concerns compared to adults, such as issues related to schooling, social interactions, and future career prospects [38]. Similarly, cultural factors may influence how patients perceive their disease, cope with symptoms, and respond to treatments [39]. Therefore, demographic-sensitive measures ensure that the unique experiences and needs of all patient groups are captured accurately. Second, accurate HRQoL assessments are crucial for assessing the impact of HAE on patients’ lives and monitoring changes over time [40]. Tools not adapted to specific demographic or cultural contexts may miss critical nuances, leading to incomplete or misleading assessments. Third, understanding the HRQoL issues in different demographic groups allows healthcare providers, such as physicians, nurses, and psychologists, to tailor treatment plans more effectively. Personalized treatment plans addressing the unique needs of each patient group help to improve adherence to therapies and overall health outcomes, as demonstrated, for instance, in people with diabetes [41].

Although this review makes an important contribution to the literature, a few important limitations remain. Most importantly, the PROMs for HAE have not been subjected to in-depth validation in content validity and responsiveness. Content validity would provide evidence that the instrument is measuring all relevant aspects of HRQoL from the perspective of the patient, while responsiveness would describe how well the instrument could detect clinically important change over time. The lack of comprehensive evaluation of these types leads to incomplete evidence on HRQOL effects. Additionally, the data included in this review may be limited by potential publication bias, as only published studies were included, which may over-represent positive findings. Another limitation of the systematic review process is the possibility of selection bias, particularly given the exclusion of articles in languages other than English and the reliance on specific databases. These factors could result in the underrepresentation of certain cultural or geographic contexts. Furthermore, the heterogeneity in study designs, sample sizes, and measurement properties reported across the included studies limited our ability to perform quantitative synthesis. These limitations underscore the need for further research to validate PROMs comprehensively and include diverse linguistic and cultural populations to improve generalizability.

Future research should, therefore, focus on how to address these limitations effectively. Such research studies must be performed with varied groups of patients, including younger patients and those from various cultural backgrounds. This would make the development of new or revised PROMs easier to capture in the lived experience of all HAE patients. The design of longitudinal studies also involves the use of follow-up scores and change scores over time in patients’ HRQoL, particularly if a treatment intervention has been introduced to detect significant clinical changes with responsive features of PROMs.

## Conclusions

The current systematic review has highlighted an important gap in the evaluation of HRQoL tools for HAE, identifying the strengths and limitations of existing PROMs. While the review identifies several generic and disease-specific tools, it underscores the need for more comprehensive validation, especially concerning cross-cultural contexts and younger populations of the current measures and the devolution of refined versions of disease-specific tools to overcome their current limitations. Furthermore, the review highlights the need for studies confirming measurement invariance across different cultural and language contexts for the available HRQoL tools. Priorities for future research include enhancing content validity and responsiveness of PROMs, developing age-specific tools, and ensuring validation across diverse cultural and linguistic groups.

## Supplementary Information

Supplementary Material 1.

Supplementary Material 2.

Supplementary Material 3.

Supplementary Material 4.

Supplementary Material 5.

Supplementary Material 6.

## Data Availability

No datasets were generated or analysed during the current study.

## Abbreviations

AE-QoL: Angioedema Quality of Life Questionnaire
COSMIN: COnsensus-based Standards for the selection of health Measurement INstruments
C1-INH: C1-inhibitor
GRADE: Grading of Recommendations Assessment, Development and Evaluation
HAE: Hereditary Angioedema
HAE-C1-INH: Hereditary Angioedema due to C1-inhibitor deficiency
HAE-QoL: Hereditary Angioedema Quality of Life
HAEA-QoL: Hereditary Angioedema Association Quality of Life
HRQoL: Health-Related Quality of Life
ICC: Intraclass Correlation
LTP: Long-Term Prophylaxis
PROM: Patient-Reported Outcome Measure
PRISMA: Preferred Reporting Items for Systematic Reviews and Meta-Analyses
PROSPERO: International Prospective Register of Systematic Reviews
SDS: Sheehan Disability Scale
SF-36: Short Form 36-item Health Survey
SF-36v2: Short Form 36-item Health Survey Version 2.0
VAS: Visual Analog Scale
