# Supplementary figures and images for: A COSMIN systematic review of instruments for evaluating health-related quality of life in people with Hereditary Angioedema
Source: Health Qual Life Outcomes. 2025 Feb 13;23:12. doi: 10.1186/s12955-025-02342-6 (PMC11823193; doi:10.1186/s12955-025-02342-6)

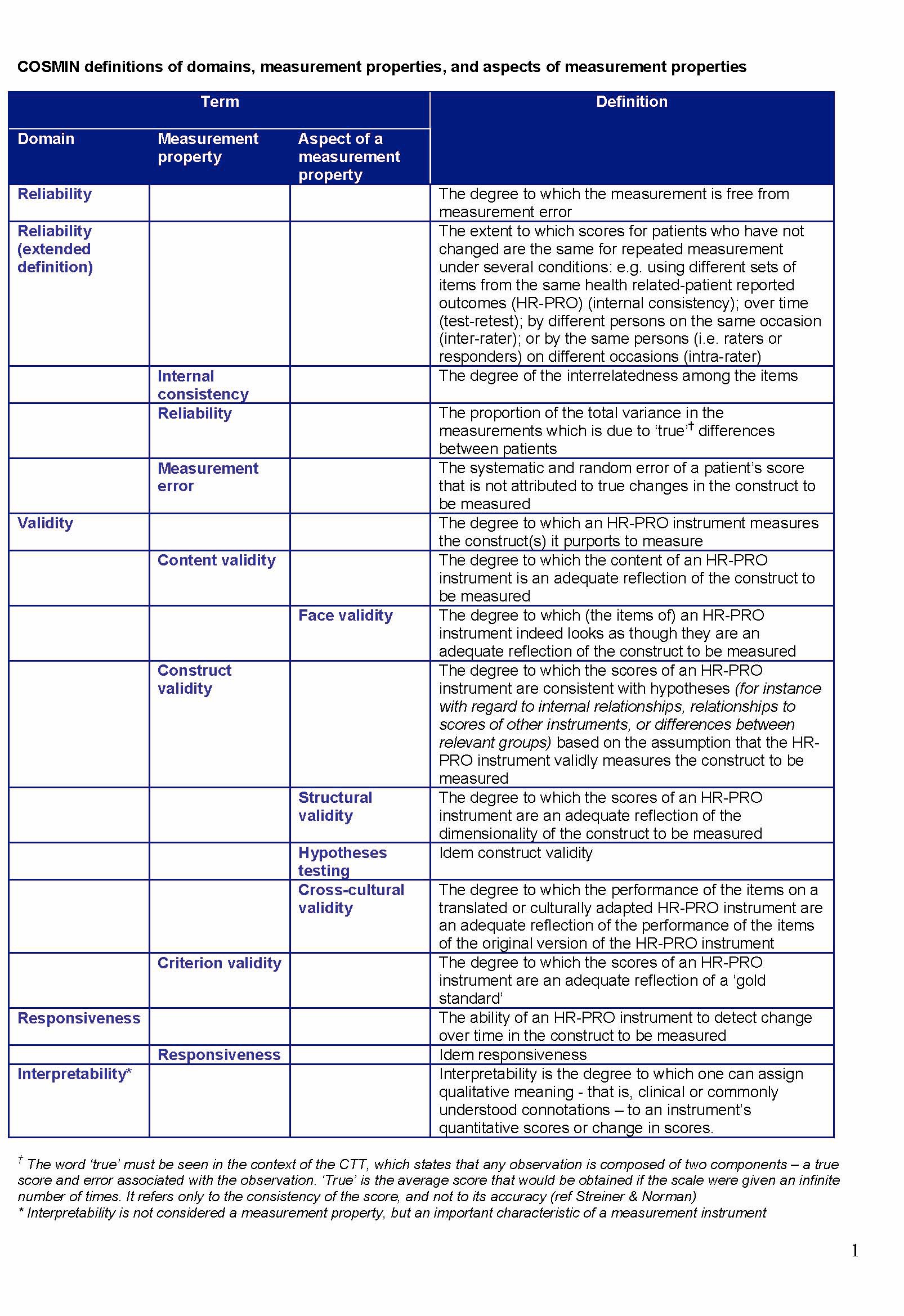

Supplement: Supplementary file 2 — Supplementary Material 2. [file 12955_2025_2342_MOESM2_ESM.docx]
