# Supplementary material for: A COSMIN systematic review of instruments for evaluating health-related quality of life in people with Hereditary Angioedema
Source: Health Qual Life Outcomes. 2025 Feb 13;23:12. doi: 10.1186/s12955-025-02342-6 (PMC11823193; doi:10.1186/s12955-025-02342-6)
Supplement: Supplementary file 3 — Supplementary Material 3. [file 12955_2025_2342_MOESM3_ESM.pdf]

A COSMIN systematic review of instruments used to evaluate Health-related quality of life of people with hereditary angioedema (HAE)

AIM: The purpose of the review is to identify, analyse and summarise the critical aspects of all the tools present in the scientific literature that measure the health-related quality of life in people with hereditary angioedema (HAE).

QUESTION: What are the most appropriate, valid and reliable instruments to assess the health-related quality of life in people with hereditary angioedema (HAE) (any type)?

RESEARCH QUESTION WITH COSMIN METHOD

1) **Population:** in people (any age) with hereditary angioedema (HAE) (any type)
2) type of **Instruments:** Any type of instrument (ie questionnaire, inventory) and all patient-reported outcome measures (PROMs)
3) **Construct:** Health-related quality of life
4) **Outcome/Measurement properties:** all measurement properties (i.e. structural validity, internal consistency, cross-cultural validity\measurement invariance, reliability, measurement error, criterion validity, hypotheses testing for construct validity, and responsiveness)

Methods

The sources that will be searched are PubMed; Scopus; Web of Science; Embase; CINAHL. No time/language restrictions will be applied.

Population

Pubmed:

"Angioedemas, Hereditary"[Mesh] OR "Hereditary Angioedema"[tw] OR "Hereditary Angioedemas"[tw] OR "Angioedema, Hereditary"[tw] OR "Hereditary angioneurotic edema"[tw] OR "familial angioneurotic edema"[tw] OR "Hereditary angioneurotic oedema"[tw]

Scopus

INDEXTERMS("Angioedemas, Hereditary") OR TITLE-ABS-KEY("Hereditary Angioedema") OR TITLE-ABS-KEY("Hereditary Angioedemas") OR TITLE-ABS-KEY("Angioedema, Hereditary") OR TITLE-ABS-KEY("Hereditary angioneurotic edema") OR TITLE-ABS-KEY("familial angioneurotic edema") OR TITLE-ABS-KEY("Hereditary angioneurotic oedema")

Web of Science

ALL="Angioedemas, Hereditary" OR ALL="Hereditary Angioedema" OR ALL="Hereditary Angioedemas" OR ALL="Angioedema, Hereditary" OR ALL="Hereditary angioneurotic edema" OR ALL="familial angioneurotic edema" OR ALL="Hereditary angioneurotic oedema"

Embase

'Angioedemas, Hereditary'/exp OR 'Hereditary Angioedema' OR 'Hereditary Angioedemas' OR 'Angioedema, Hereditary' OR 'Hereditary angioneurotic edema' OR 'familial angioneurotic edema' OR 'Hereditary angioneurotic oedema'

CINAHL

(MH "Angioedemas, Hereditary+") OR "Hereditary Angioedema" OR "Hereditary Angioedemas" OR "Angioedema, Hereditary" OR "Hereditary angioneurotic edema" OR "familial angioneurotic edema" OR "Hereditary angioneurotic oedema"

type of Instruments: No specific query to keep broad the identification phase.

Construct

Pubmed:

"Quality of Life"[Mesh] OR "Life Quality"[tw] OR "Health Related Quality Of Life"[tw] OR "Health-Related Quality Of Life"[tw] OR HRQOL[tw] OR QoL[tw]

Scopus

INDEXTERMS("Quality of Life") OR TITLE-ABS-KEY("Life Quality") OR TITLE-ABS-KEY("Health Related Quality Of Life") OR TITLE-ABS-KEY("Health-Related Quality Of Life") OR TITLE-ABS-KEY(HRQOL) OR TITLE-ABS-KEY(QoL)

Web of Science

ALL="Quality of Life" OR ALL="Life Quality" OR ALL="Health Related Quality Of Life" OR ALL="Health-Related Quality Of Life" OR ALL=HRQOL OR ALL=QoL

Embase

'Quality of Life'/exp OR 'Life Quality' OR 'Health Related Quality Of Life' OR 'Health-Related Quality Of Life' OR HRQOL OR QoL

CINAHL

(MH "Quality of Life+") OR "Life Quality" OR "Health Related Quality Of Life" OR "Health-Related Quality Of Life" OR HRQOL OR QoL

Outcome/Measurement properties[1]

Pubmed:

(instrumentation[sh] OR methods[sh] OR “Validation Studies”[pt] OR “Comparative Study”[pt] OR “psychometrics”[MeSH] OR psychometr*[tiab] OR clinimetr*[tw] OR clinometr*[tw] OR “outcome assessment (health care)”[MeSH] OR “outcome assessment”[tiab] OR “outcome measure*”[tw] OR “observer variation”[MeSH] OR “observer variation”[tiab] OR “Health Status Indicators”[Mesh] OR “reproducibility of results”[MeSH] OR reproducib*[tiab] OR “discriminant analysis”[MeSH] OR reliab*[tiab] OR unreliab*[tiab] OR valid*[tiab] OR “coefficient of variation”[tiab] OR coefficient[tiab] OR homogeneity[tiab] OR homogeneous[tiab] OR “internal consistency”[tiab] OR (cronbach*[tiab] AND (alpha[tiab] OR alphas[tiab])) OR (item[tiab] AND (correlation*[tiab] OR selection*[tiab] OR reduction*[tiab])) OR agreement[tw] OR precision[tw] OR imprecision[tw] OR “precise values”[tw] OR test-

[1] Terwee CB, Jansma EP, Riphagen II, de Vet HC. Development of a methodological PubMed search filter for finding studies on measurement properties of measurement instruments. Qual Life Res. 2009 Oct;18(8):1115-23. doi: 10.1007/s11136-009-9528-5.

retest[tiab] OR (test[tiab] AND retest[tiab]) OR (reliab*[tiab] AND (test[tiab] OR retest[tiab])) OR stability[tiab] OR interrater[tiab] OR inter-rater[tiab] OR intrarater[tiab] OR intra-rater[tiab] OR intertester[tiab] OR inter-tester[tiab] OR intratester[tiab] OR intra-tester[tiab] OR interobserver[tiab] OR inter-observer[tiab] OR intraobserver[tiab] OR intra-observer[tiab] OR intertechnician[tiab] OR inter-technician[tiab] OR intratechnician[tiab] OR intra-technician[tiab] OR interexaminer[tiab] OR inter-examiner[tiab] OR intraexaminer[tiab] OR intra-examiner[tiab] OR interassay[tiab] OR inter-assay[tiab] OR intraassay[tiab] OR intra-assay[tiab] OR interindividual[tiab] OR inter-individual[tiab] OR intraindividual[tiab] OR intra-individual[tiab] OR interparticipant[tiab] OR inter-participant[tiab] OR intraparticipant[tiab] OR intra-participant[tiab] OR kappa[tiab] OR kappa’s[tiab] OR kappas[tiab] OR repeatab*[tw] OR ((replicab*[tw] OR repeated[tw]) AND (measure[tw] OR measures[tw] OR findings[tw] OR result[tw] OR results[tw] OR test[tw] OR tests[tw])) OR generaliza*[tiab] OR generalisa*[tiab] OR concordance[tiab] OR (intraclass[tiab] AND correlation*[tiab]) OR discriminative[tiab] OR “known group”[tiab] OR “factor analysis”[tiab] OR “factor analyses”[tiab] OR “factor structure”[tiab] OR “factor structures”[tiab] OR dimension*[tiab] OR subscale*[tiab] OR (multitrait[tiab] AND scaling[tiab] AND (analysis[tiab] OR analyses[tiab])) OR “item discriminant”[tiab] OR “interscale correlation*”[tiab] OR error[tiab] OR errors[tiab] OR “individual variability”[tiab] OR “interval variability”[tiab] OR “rate variability”[tiab] OR (variability[tiab] AND (analysis[tiab] OR values[tiab])) OR (uncertainty[tiab] AND (measurement[tiab] OR measuring[tiab])) OR “standard error of measurement”[tiab] OR sensitiv*[tiab] OR responsive*[tiab] OR (limit[tiab] AND detection[tiab]) OR “minimal detectable concentration”[tiab] OR interpretab*[tiab] OR ((minimal[tiab] OR minimally[tiab] OR clinical[tiab] OR clinically[tiab]) AND (important[tiab] OR significant[tiab] OR detectable[tiab]) AND (change[tiab] OR difference[tiab])) OR (small*[tiab] AND (real[tiab] OR detectable[tiab]) AND (change[tiab] OR difference[tiab])) OR “meaningful change”[tiab] OR “ceiling effect”[tiab] OR “floor effect”[tiab] OR “Item response model”[tiab] OR IRT[tiab] OR Rasch[tiab] OR “Differential item functioning”[tiab] OR DIF[tiab] OR “computer adaptive testing”[tiab] OR “item bank”[tiab] OR “cross-cultural equivalence”[tiab])

Scopus

("Instrumentation" OR "Methods" OR DOCTYPE("Validation Studies") OR DOCTYPE("Comparative Study") OR INDEXTERMS(psychometrics) OR TITLE-ABS(psychometr*) OR TITLE-ABS-KEY(clinimetr*) OR TITLE-ABS-KEY(clinometr*) OR INDEXTERMS("outcome assessment (health care)") OR TITLE-ABS("outcome assessment") OR TITLE-ABS-KEY("outcome measure*") OR INDEXTERMS("observer variation") OR TITLE-ABS("observer variation") OR INDEXTERMS("Health Status Indicators") OR INDEXTERMS("reproducibility of results") OR TITLE-ABS(reproducib*) OR INDEXTERMS("discriminant analysis") OR TITLE-ABS(reliab*) OR TITLE-ABS(unreliab*) OR TITLE-ABS(valid*) OR TITLE-ABS("coefficient of variation") OR TITLE-ABS(coefficient) OR TITLE-ABS(homogeneity) OR TITLE-ABS(homogeneous) OR TITLE-ABS("internal consistency") OR (TITLE-ABS(cronbach*) AND (TITLE-ABS(alpha) OR TITLE-ABS(alphas))) OR (TITLE-ABS(item) AND (TITLE-ABS(correlation*) OR TITLE-ABS(selection*) OR TITLE-ABS(reduction*))) OR TITLE-ABS-KEY(agreement) OR TITLE-ABS-KEY(precision) OR TITLE-ABS-KEY(imprecision) OR TITLE-ABS-KEY("precise values") OR TITLE-ABS(test-retest) OR (TITLE-ABS(test) AND TITLE-ABS(retest)) OR (TITLE-ABS(reliab*) AND (TITLE-ABS(test) OR TITLE-ABS(retest))) OR TITLE-ABS(stability) OR TITLE-ABS(interrater) OR TITLE-ABS(inter-rater) OR TITLE-ABS(intrarater) OR TITLE-ABS(intra-rater) OR TITLE-ABS(intertester) OR TITLE-ABS(inter-tester) OR TITLE-ABS(intratester) OR TITLE-ABS(intra-tester) OR TITLE-ABS(interobserver) OR TITLE-ABS(inter-observer) OR TITLE-ABS(intraobserver) OR TITLE-ABS(intra-observer) OR TITLE-ABS(intertechnician) OR TITLE-ABS(inter-technician) OR TITLE-ABS(intratechnician) OR TITLE-ABS(intra-technician) OR TITLE-ABS(interexaminer) OR TITLE-ABS(inter-examiner) OR TITLE-ABS(intraexaminer) OR TITLE-ABS(intra-examiner) OR TITLE-ABS(interassay) OR TITLE-ABS(inter-assay) OR TITLE-ABS(intraassay) OR TITLE-ABS(intra-assay) OR TITLE-ABS(interindividual) OR TITLE-ABS(inter-individual) OR TITLE-ABS(intraindividual) OR TITLE-ABS(intra-individual) OR TITLE-ABS(interparticipant) OR TITLE-ABS(inter-participant) OR TITLE-ABS(intraparticipant) OR TITLE-ABS(intra-participant) OR TITLE-ABS(kappa) OR TITLE-ABS(kappa’s) OR TITLE-ABS(kappas) OR TITLE-ABS-KEY(repeatab*) OR ((TITLE-ABS-KEY(replicab*) OR TITLE-ABS-KEY(repeated)) AND (TITLE-ABS-KEY(measure) OR TITLE-ABS-KEY(measures) OR TITLE-ABS-KEY(findings) OR TITLE-ABS-KEY(result) OR TITLE-ABS-KEY(results) OR TITLE-ABS-KEY(test) OR TITLE-ABS-KEY(tests))) OR TITLE-ABS(generaliza*) OR TITLE-

ABS(generalisa*) OR TITLE-ABS(concordance) OR (TITLE-ABS(intraclass) AND TITLE-ABS(correlation*)) OR TITLE-ABS(discriminative) OR TITLE-ABS("known group") OR TITLE-ABS("factor analysis") OR TITLE-ABS("factor analyses") OR TITLE-ABS("factor structure") OR TITLE-ABS("factor structures") OR TITLE-ABS(dimension*) OR TITLE-ABS(subscale*) OR (TITLE-ABS(multitrait) AND TITLE-ABS(scaling) AND (TITLE-ABS(analysis) OR TITLE-ABS(analyses))) OR TITLE-ABS("item discriminant") OR TITLE-ABS("interscale correlation*") OR TITLE-ABS(error) OR TITLE-ABS(errors) OR TITLE-ABS("individual variability") OR TITLE-ABS("interval variability") OR TITLE-ABS("rate variability") OR (TITLE-ABS(variability) AND (TITLE-ABS(analysis) OR TITLE-ABS(values))) OR (TITLE-ABS(uncertainty) AND (TITLE-ABS(measurement) OR TITLE-ABS(measuring))) OR TITLE-ABS("standard error of measurement") OR TITLE-ABS(sensitiv*) OR TITLE-ABS(responsive*) OR (TITLE-ABS(limit) AND TITLE-ABS(detection)) OR TITLE-ABS("minimal detectable concentration") OR TITLE-ABS(interpretab*) OR ((TITLE-ABS(minimal) OR TITLE-ABS(minimally) OR TITLE-ABS(clinical) OR TITLE-ABS(clinically)) AND (TITLE-ABS(important) OR TITLE-ABS(significant) OR TITLE-ABS(detectable)) AND (TITLE-ABS(change) OR TITLE-ABS(difference))) OR (TITLE-ABS(small*) AND (TITLE-ABS(real) OR TITLE-ABS(detectable)) AND (TITLE-ABS(change) OR TITLE-ABS(difference))) OR TITLE-ABS("meaningful change") OR TITLE-ABS("ceiling effect") OR TITLE-ABS("floor effect") OR TITLE-ABS("Item response model") OR TITLE-ABS(IRT) OR TITLE-ABS(Rasch) OR TITLE-ABS("Differential item functioning") OR TITLE-ABS(DIF) OR TITLE-ABS("computer adaptive testing") OR TITLE-ABS("item bank") OR TITLE-ABS("cross-cultural equivalence"))

Web of Science

(ALL="Instrumentation" OR ALL="Methods" OR ALL="Validation Studies" OR ALL="Comparative Study" OR ALL=psychometrics OR (TI=psychometr* OR AB=psychometr*) OR ALL=clinimetr* OR ALL=clinometr* OR ALL="outcome assessment (health care)" OR (TI="outcome assessment" OR AB="outcome assessment") OR ALL="outcome measure*" OR ALL="observer variation" OR (TI="observer variation" OR AB="observer variation") OR ALL="Health Status Indicators" OR ALL="reproducibility of results" OR (TI=reproducib* OR AB=reproducib*) OR ALL="discriminant analysis" OR (TI=reliab* OR AB=reliab*) OR (TI=unreliab* OR AB=unreliab*) OR (TI=valid* OR AB=valid*) OR (TI="coefficient of variation" OR AB="coefficient of variation") OR (TI=coefficient OR AB=coefficient) OR (TI=homogeneity OR AB=homogeneity) OR (TI=homogeneous OR AB=homogeneous) OR (TI="internal consistency" OR AB="internal consistency") OR ((TI=cronbach* OR AB=cronbach*) AND ((TI=alpha OR AB=alpha) OR (TI=alphas OR AB=alphas))) OR ((TI=item OR AB=item) AND ((TI=correlation* OR AB=correlation*) OR (TI=selection* OR AB=selection*) OR (TI=reduction* OR AB=reduction*))) OR ALL=agreement OR ALL=precision OR ALL=imprecision OR ALL="precise values" OR (TI=test-retest OR AB=test-retest) OR ((TI=test OR AB=test) AND (TI=retest OR AB=retest)) OR ((TI=reliab* OR AB=reliab*) AND ((TI=test OR AB=test) OR (TI=retest OR AB=retest))) OR (TI=stability OR AB=stability) OR (TI=interrater OR AB=interrater) OR (TI=inter-rater OR AB=inter-rater) OR (TI=intrarater OR AB=intrarater) OR (TI=intra-rater OR AB=intra-rater) OR (TI=intertester OR AB=intertester) OR (TI=inter-tester OR AB=inter-tester) OR (TI=intratester OR AB=intratester) OR (TI=intra-tester OR AB=intra-tester) OR (TI=interobserver OR AB=interobserver) OR (TI=inter-observer OR AB=inter-observer) OR (TI=intraobserver OR AB=intraobserver) OR (TI=intra-observer OR AB=intra-observer) OR (TI=intertechnician OR AB=intertechnician) OR (TI=inter-technician OR AB=inter-technician) OR (TI=intratechnician OR AB=intratechnician) OR (TI=intra-technician OR AB=intra-technician) OR (TI=interexaminer OR AB=interexaminer) OR (TI=inter-examiner OR AB=inter-examiner) OR (TI=intraexaminer OR AB=intraexaminer) OR (TI=intra-examiner OR AB=intra-examiner) OR (TI=interassay OR AB=interassay) OR (TI=inter-assay OR AB=inter-assay) OR (TI=intraassay OR AB=intraassay) OR (TI=intra-assay OR AB=intra-assay) OR (TI=interindividual OR AB=interindividual) OR (TI=inter-individual OR AB=inter-individual) OR (TI=intraindividual OR AB=intraindividual) OR (TI=intra-individual OR AB=intra-individual) OR (TI=interparticipant OR AB=interparticipant) OR (TI=inter-participant OR AB=inter-participant) OR (TI=intraparticipant OR AB=intraparticipant) OR (TI=intra-participant OR AB=intra-participant) OR (TI=kappa OR AB=kappa) OR (TI=kappa's OR AB=kappa's) OR (TI=kappas OR AB=kappas) OR ALL=repeatab* OR ((ALL=replicab* OR ALL=repeated) AND (ALL=measure OR ALL=measures OR ALL=findings OR ALL=result OR ALL=results OR ALL=test OR ALL=tests)) OR (TI=generaliza* OR AB=generaliza*) OR (TI=generalisa* OR AB=generalisa*) OR (TI=concordance OR AB=concordance) OR ((TI=intraclass OR AB=intraclass) AND

(TI=correlation* OR AB=correlation*)) OR (TI=discriminative OR AB=discriminative) OR (TI="known group" OR AB="known group") OR (TI="factor analysis" OR AB="factor analysis") OR (TI="factor analyses" OR AB="factor analyses") OR (TI="factor structure" OR AB="factor structure") OR (TI="factor structures" OR AB="factor structures") OR (TI=dimension* OR AB=dimension*) OR (TI=subscale* OR AB=subscale*) OR ((TI=multitrait OR AB=multitrait) AND (TI=scaling OR AB=scaling) AND ((TI=analysis OR AB=analysis) OR (TI=analyses OR AB=analyses))) OR (TI="item discriminant" OR AB="item discriminant") OR (TI="interscale correlation*" OR AB="interscale correlation*") OR (TI=error OR AB=error) OR (TI=errors OR AB=errors) OR (TI="individual variability" OR AB="individual variability") OR (TI="interval variability" OR AB="interval variability") OR (TI="rate variability" OR AB="rate variability") OR ((TI=variability OR AB=variability) AND ((TI=analysis OR AB=analysis) OR (TI=values OR AB=values))) OR ((TI=uncertainty OR AB=uncertainty) AND ((TI=measurement OR AB=measurement) OR (TI=measuring OR AB=measuring))) OR (TI="standard error of measurement" OR AB="standard error of measurement") OR (TI=sensitiv* OR AB=sensitiv*) OR (TI=responsive* OR AB=responsive*) OR ((TI=limit OR AB=limit) AND (TI=detection OR AB=detection)) OR (TI="minimal detectable concentration" OR AB="minimal detectable concentration") OR (TI=interpretab* OR AB=interpretab*) OR (((TI=minimal OR AB=minimal) OR (TI=minimally OR AB=minimally) OR (TI=clinical OR AB=clinical) OR (TI=clinically OR AB=clinically)) AND ((TI=important OR AB=important) OR (TI=significant OR AB=significant) OR (TI=detectable OR AB=detectable)) AND ((TI=change OR AB=change) OR (TI=difference OR AB=difference))) OR ((TI=small* OR AB=small*) AND ((TI=real OR AB=real) OR (TI=detectable OR AB=detectable)) AND ((TI=change OR AB=change) OR (TI=difference OR AB=difference))) OR (TI="meaningful change" OR AB="meaningful change") OR (TI="ceiling effect" OR AB="ceiling effect") OR (TI="floor effect" OR AB="floor effect") OR (TI="Item response model" OR AB="Item response model") OR (TI=IRT OR AB=IRT) OR (TI=Rasch OR AB=Rasch) OR (TI="Differential item functioning" OR AB="Differential item functioning") OR (TI=DIF OR AB=DIF) OR (TI="computer adaptive testing" OR AB="computer adaptive testing") OR (TI="item bank" OR AB="item bank") OR (TI="cross-cultural equivalence" OR AB="cross-cultural equivalence"))

Embase

("Instrumentation" OR "Methods" OR term:it OR term:it OR psychometrics/exp OR psychometr*:ti,ab OR clinimetr* OR clinometr* OR 'outcome assessment (health care)'/exp OR 'outcome assessment':ti,ab OR 'outcome measure*' OR 'observer variation'/exp OR 'observer variation':ti,ab OR 'Health Status Indicators'/exp OR 'reproducibility of results'/exp OR reproducib*:ti,ab OR 'discriminant analysis'/exp OR reliab*:ti,ab OR unreliab*:ti,ab OR valid*:ti,ab OR 'coefficient of variation':ti,ab OR coefficient:ti,ab OR homogeneity:ti,ab OR homogeneous:ti,ab OR 'internal consistency':ti,ab OR (cronbach*:ti,ab AND (alpha:ti,ab OR alphas:ti,ab)) OR (item:ti,ab AND (correlation*:ti,ab OR selection*:ti,ab OR reduction*:ti,ab)) OR agreement OR precision OR imprecision OR 'precise values' OR test-retest:ti,ab OR (test:ti,ab AND retest:ti,ab) OR (reliab*:ti,ab AND (test:ti,ab OR retest:ti,ab)) OR stability:ti,ab OR interrater:ti,ab OR inter-rater:ti,ab OR intrarater:ti,ab OR intra-rater:ti,ab OR intertester:ti,ab OR inter-tester:ti,ab OR intratester:ti,ab OR intra-tester:ti,ab OR interobserver:ti,ab OR inter-observer:ti,ab OR intraobserver:ti,ab OR intra-observer:ti,ab OR intertechnician:ti,ab OR inter-technician:ti,ab OR intratechnician:ti,ab OR intra-technician:ti,ab OR interexaminer:ti,ab OR inter-examiner:ti,ab OR intraexaminer:ti,ab OR intra-examiner:ti,ab OR interassay:ti,ab OR inter-assay:ti,ab OR intraassay:ti,ab OR intra-assay:ti,ab OR interindividual:ti,ab OR inter-individual:ti,ab OR intraindividual:ti,ab OR intra-individual:ti,ab OR interparticipant:ti,ab OR inter-participant:ti,ab OR intraparticipant:ti,ab OR intra-participant:ti,ab OR kappa:ti,ab OR kappa’s:ti,ab OR kappas:ti,ab OR repeatab* OR ((replicab* OR repeated) AND (measure OR measures OR findings OR result OR results OR test OR tests)) OR generaliza*:ti,ab OR generalisa*:ti,ab OR concordance:ti,ab OR (intraclass:ti,ab AND correlation*:ti,ab) OR discriminative:ti,ab OR 'known group':ti,ab OR 'factor analysis':ti,ab OR 'factor analyses':ti,ab OR 'factor structure':ti,ab OR 'factor structures':ti,ab OR dimension*:ti,ab OR subscale*:ti,ab OR (multitrait:ti,ab AND scaling:ti,ab AND (analysis:ti,ab OR analyses:ti,ab)) OR 'item discriminant':ti,ab OR 'interscale correlation*':ti,ab OR error:ti,ab OR errors:ti,ab OR 'individual variability':ti,ab OR 'interval variability':ti,ab OR 'rate variability':ti,ab OR (variability:ti,ab AND (analysis:ti,ab OR values:ti,ab)) OR (uncertainty:ti,ab AND (measurement:ti,ab OR measuring:ti,ab)) OR 'standard error of measurement':ti,ab OR sensitiv*:ti,ab OR responsive*:ti,ab OR (limit:ti,ab AND detection:ti,ab) OR 'minimal detectable concentration':ti,ab OR interpretab*:ti,ab OR ((minimal:ti,ab OR

minimally:ti,ab OR clinical:ti,ab OR clinically:ti,ab) AND (important:ti,ab OR significant:ti,ab OR detectable:ti,ab) AND (change:ti,ab OR difference:ti,ab)) OR (small*:ti,ab AND (real:ti,ab OR detectable:ti,ab) AND (change:ti,ab OR difference:ti,ab)) OR 'meaningful change':ti,ab OR 'ceiling effect':ti,ab OR 'floor effect':ti,ab OR 'Item response model':ti,ab OR IRT:ti,ab OR Rasch:ti,ab OR 'Differential item functioning':ti,ab OR DIF:ti,ab OR 'computer adaptive testing':ti,ab OR 'item bank':ti,ab OR 'cross-cultural equivalence':ti,ab)

CINAHL

("Instrumentation" OR "Methods" OR (PT "Validation Studies") OR (PT "Comparative Study") OR (MH psychometrics+) OR (TI psychometr* OR AB psychometr*) OR clinimetr* OR clinometr* OR (MH "outcome assessment (health care)+") OR (TI "outcome assessment" OR AB "outcome assessment") OR "outcome measure*" OR (MH "observer variation+") OR (TI "observer variation" OR AB "observer variation") OR (MH "Health Status Indicators+") OR (MH "reproducibility of results+") OR (TI reproducib* OR AB reproducib*) OR (MH "discriminant analysis+") OR (TI reliab* OR AB reliab*) OR (TI unreliab* OR AB unreliab*) OR (TI valid* OR AB valid*) OR (TI "coefficient of variation" OR AB "coefficient of variation") OR (TI coefficient OR AB coefficient) OR (TI homogeneity OR AB homogeneity) OR (TI homogeneous OR AB homogeneous) OR (TI "internal consistency" OR AB "internal consistency") OR ((TI cronbach* OR AB cronbach*) AND ((TI alpha OR AB alpha) OR (TI alphas OR AB alphas))) OR ((TI item OR AB item) AND ((TI correlation* OR AB correlation*) OR (TI selection* OR AB selection*) OR (TI reduction* OR AB reduction*))) OR agreement OR precision OR imprecision OR "precise values" OR (TI test-retest OR AB test-retest) OR ((TI test OR AB test) AND (TI retest OR AB retest)) OR ((TI reliab* OR AB reliab*) AND ((TI test OR AB test) OR (TI retest OR AB retest))) OR (TI stability OR AB stability) OR (TI interrater OR AB interrater) OR (TI inter-rater OR AB inter-rater) OR (TI intrarater OR AB intrarater) OR (TI intra-rater OR AB intra-rater) OR (TI intertester OR AB intertester) OR (TI inter-tester OR AB inter-tester) OR (TI intratester OR AB intratester) OR (TI intra-tester OR AB intra-tester) OR (TI interobserver OR AB interobserver) OR (TI inter-observer OR AB inter-observer) OR (TI intraobserver OR AB intraobserver) OR (TI intra-observer OR AB intra-observer) OR (TI intertechnician OR AB intertechnician) OR (TI inter-technician OR AB inter-technician) OR (TI intratechnician OR AB intratechnician) OR (TI intra-technician OR AB intra-technician) OR (TI interexaminer OR AB interexaminer) OR (TI inter-examiner OR AB inter-examiner) OR (TI intraexaminer OR AB intraexaminer) OR (TI intra-examiner OR AB intra-examiner) OR (TI interassay OR AB interassay) OR (TI inter-assay OR AB inter-assay) OR (TI intraassay OR AB intraassay) OR (TI intra-assay OR AB intra-assay) OR (TI interindividual OR AB interindividual) OR (TI inter-individual OR AB inter-individual) OR (TI intraindividual OR AB intraindividual) OR (TI intra-individual OR AB intra-individual) OR (TI interparticipant OR AB interparticipant) OR (TI inter-participant OR AB inter-participant) OR (TI intraparticipant OR AB intraparticipant) OR (TI intra-participant OR AB intra-participant) OR (TI kappa OR AB kappa) OR (TI kappa's OR AB kappa's) OR (TI kappas OR AB kappas) OR repeatab* OR ((replicab* OR repeated) AND (measure OR measures OR findings OR result OR results OR test OR tests)) OR (TI generaliza* OR AB generaliza*) OR (TI generalisa* OR AB generalisa*) OR (TI concordance OR AB concordance) OR ((TI intraclass OR AB intraclass) AND (TI correlation* OR AB correlation*)) OR (TI discriminative OR AB discriminative) OR (TI "known group" OR AB "known group") OR (TI "factor analysis" OR AB "factor analysis") OR (TI "factor analyses" OR AB "factor analyses") OR (TI "factor structure" OR AB "factor structure") OR (TI "factor structures" OR AB "factor structures") OR (TI dimension* OR AB dimension*) OR (TI subscale* OR AB subscale*) OR ((TI multitrait OR AB multitrait) AND (TI scaling OR AB scaling) AND ((TI analysis OR AB analysis) OR (TI analyses OR AB analyses))) OR (TI "item discriminant" OR AB "item discriminant") OR (TI "interscale correlation*" OR AB "interscale correlation*") OR (TI error OR AB error) OR (TI errors OR AB errors) OR (TI "individual variability" OR AB "individual variability") OR (TI "interval variability" OR AB "interval variability") OR (TI "rate variability" OR AB "rate variability") OR ((TI variability OR AB variability) AND ((TI analysis OR AB analysis) OR (TI values OR AB values))) OR ((TI uncertainty OR AB uncertainty) AND ((TI measurement OR AB measurement) OR (TI measuring OR AB measuring))) OR (TI "standard error of measurement" OR AB "standard error of measurement") OR (TI sensitiv* OR AB sensitiv*) OR (TI responsive* OR AB responsive*) OR ((TI limit OR AB limit) AND (TI detection OR AB detection)) OR (TI "minimal detectable concentration" OR AB "minimal detectable concentration") OR (TI interpretab* OR AB interpretab*) OR (((TI minimal OR AB minimal) OR (TI minimally OR AB minimally) OR (TI clinical OR AB clinical) OR (TI clinically OR AB clinically)) AND ((TI important OR AB important) OR (TI significant OR AB significant)

OR (TI detectable OR AB detectable)) AND ((TI change OR AB change) OR (TI difference OR AB difference))) OR ((TI small* OR AB small*) AND ((TI real OR AB real) OR (TI detectable OR AB detectable)) AND ((TI change OR AB change) OR (TI difference OR AB difference))) OR (TI "meaningful change" OR AB "meaningful change") OR (TI "ceiling effect" OR AB "ceiling effect") OR (TI "floor effect" OR AB "floor effect") OR (TI "Item response model" OR AB "Item response model") OR (TI IRT OR AB IRT) OR (TI Rasch OR AB Rasch) OR (TI "Differential item functioning" OR AB "Differential item functioning") OR (TI DIF OR AB DIF) OR (TI "computer adaptive testing" OR AB "computer adaptive testing") OR (TI "item bank" OR AB "item bank") OR (TI "cross-cultural equivalence" OR AB "cross-cultural equivalence"))

FINAL Queries

Pubmed (99 records; 11 May)

("Angioedemas, Hereditary"[Mesh] OR "Hereditary Angioedema"[tw] OR "Hereditary Angioedemas"[tw] OR "Angioedema, Hereditary"[tw] OR "Hereditary angioneurotic edema"[tw] OR "familial angioneurotic edema"[tw] OR "Hereditary angioneurotic oedema"[tw]) AND ("Quality of Life"[Mesh] OR "Life Quality"[tw] OR "Health Related Quality Of Life"[tw] OR "Health-Related Quality Of Life"[tw] OR HRQOL[tw] OR QoL[tw]) AND (instrumentation[sh] OR methods[sh] OR “Comparative Study”[pt] OR “psychometrics”[MeSH] OR psychometr*[tiab] OR clinimetr*[tw] OR clinometr*[tw] OR "Outcome Assessment, Health Care"[Mesh] OR “outcome assessment”[tiab] OR “outcome measure*”[tw] OR “observer variation”[MeSH] OR “observer variation”[tiab] OR “Health Status Indicators”[Mesh] OR “reproducibility of results”[MeSH] OR reproducib*[tiab] OR “discriminant analysis”[MeSH] OR reliab*[tiab] OR unreliab*[tiab] OR valid*[tiab] OR “coefficient of variation”[tiab] OR coefficient[tiab] OR homogeneity[tiab] OR homogeneous[tiab] OR “internal consistency”[tiab] OR (cronbach*[tiab] AND (alpha[tiab] OR alphas[tiab])) OR (item[tiab] AND (correlation*[tiab] OR selection*[tiab] OR reduction*[tiab])) OR agreement[tw] OR precision[tw] OR imprecision[tw] OR “precise values”[tw] OR test-retest[tiab] OR (test[tiab] AND retest[tiab]) OR (reliab*[tiab] AND (test[tiab] OR retest[tiab])) OR stability[tiab] OR interrater[tiab] OR inter-rater[tiab] OR intrarater[tiab] OR intra-rater[tiab] OR intertester[tiab] OR inter-tester[tiab] OR intratester[tiab] OR intra-tester[tiab] OR interobserver[tiab] OR inter-observer[tiab] OR intraobserver[tiab] OR intra-observer[tiab] OR intertechnician[tiab] OR inter-technician[tiab] OR intratechnician[tiab] OR intra-technician[tiab] OR interexaminer[tiab] OR inter-examiner[tiab] OR intraexaminer[tiab] OR intra-examiner[tiab] OR interassay[tiab] OR inter-assay[tiab] OR intraassay[tiab] OR intra-assay[tiab] OR interindividual[tiab] OR inter-individual[tiab] OR intraindividual[tiab] OR intra-individual[tiab] OR interparticipant[tiab] OR inter-participant[tiab] OR intraparticipant[tiab] OR intra-participant[tiab] OR kappa[tiab] OR kappa’s[tiab] OR kappas[tiab] OR repeatab*[tw] OR ((replicab*[tw] OR repeated[tw]) AND (measure[tw] OR measures[tw] OR findings[tw] OR result[tw] OR results[tw] OR test[tw] OR tests[tw])) OR generaliza*[tiab] OR generalisa*[tiab] OR concordance[tiab] OR (intraclass[tiab] AND correlation*[tiab]) OR discriminative[tiab] OR “known group”[tiab] OR “factor analysis”[tiab] OR “factor analyses”[tiab] OR “factor structure”[tiab] OR “factor structures”[tiab] OR dimension*[tiab] OR subscale*[tiab] OR (multitrait[tiab] AND scaling[tiab] AND (analysis[tiab] OR analyses[tiab])) OR “item discriminant”[tiab] OR “interscale correlation*”[tiab] OR error[tiab] OR errors[tiab] OR “individual variability”[tiab] OR “interval variability”[tiab] OR “rate variability”[tiab] OR (variability[tiab] AND (analysis[tiab] OR values[tiab])) OR (uncertainty[tiab] AND (measurement[tiab] OR measuring[tiab])) OR “standard error of measurement”[tiab] OR sensitiv*[tiab] OR responsive*[tiab] OR (limit[tiab] AND detection[tiab]) OR “minimal detectable concentration”[tiab] OR interpretab*[tiab] OR ((minimal[tiab] OR minimally[tiab] OR clinical[tiab] OR clinically[tiab]) AND (important[tiab] OR significant[tiab] OR detectable[tiab]) AND (change[tiab] OR difference[tiab])) OR (small*[tiab] AND (real[tiab] OR detectable[tiab]) AND (change[tiab] OR difference[tiab])) OR “meaningful change”[tiab] OR “ceiling effect”[tiab] OR “floor effect”[tiab] OR “Item response model”[tiab] OR IRT[tiab] OR Rasch[tiab] OR “Differential item functioning”[tiab] OR DIF[tiab] OR “computer adaptive testing”[tiab] OR “item bank”[tiab] OR “cross-cultural equivalence”[tiab])

Scopus (137 records; 11 May)

(INDEXTERMS("Angioedemas, Hereditary") OR TITLE-ABS-KEY("Hereditary Angioedema") OR TITLE-ABS-KEY("Hereditary Angioedemas") OR TITLE-ABS-KEY("Angioedema, Hereditary") OR TITLE-ABS-KEY("Hereditary angioneurotic edema") OR TITLE-ABS-KEY("familial angioneurotic edema") OR TITLE-ABS-KEY("Hereditary angioneurotic oedema")) AND (INDEXTERMS("Quality of Life") OR TITLE-ABS-KEY("Life Quality") OR TITLE-ABS-KEY("Health Related Quality Of Life") OR TITLE-ABS-KEY("Health-Related Quality Of Life") OR TITLE-ABS-KEY(HRQOL) OR TITLE-ABS-KEY(QoL)) AND (("Instrumentation" OR "Methods" OR DOCTYPE("Validation Studies") OR DOCTYPE("Comparative Study") OR INDEXTERMS(psychometrics) OR TITLE-ABS(psychometr*) OR TITLE-ABS-KEY(clinimetr*) OR TITLE-ABS-KEY(clinometr*) OR INDEXTERMS("outcome assessment, health care") OR TITLE-ABS("outcome assessment") OR TITLE-ABS-KEY("outcome measure*") OR INDEXTERMS("observer variation") OR TITLE-ABS("observer variation") OR INDEXTERMS("Health Status Indicators") OR INDEXTERMS("reproducibility of results") OR TITLE-ABS(reproducib*) OR INDEXTERMS("discriminant analysis") OR TITLE-ABS(reliab*) OR TITLE-ABS(unreliab*) OR TITLE-ABS(valid*) OR TITLE-ABS("coefficient of variation") OR TITLE-ABS(coefficient) OR TITLE-ABS(homogeneity) OR TITLE-ABS(homogeneous) OR TITLE-ABS("internal consistency") OR (TITLE-ABS(cronbach*) AND (TITLE-ABS(alpha) OR TITLE-ABS(alphas))) OR (TITLE-ABS(item) AND (TITLE-ABS(correlation*) OR TITLE-ABS(selection*) OR TITLE-ABS(reduction*))) OR TITLE-ABS-KEY(agreement) OR TITLE-ABS-KEY(precision) OR TITLE-ABS-KEY(imprecision) OR TITLE-ABS-KEY("precise values") OR TITLE-ABS(test-retest) OR (TITLE-ABS(test) AND TITLE-ABS(retest)) OR (TITLE-ABS(reliab*) AND (TITLE-ABS(test) OR TITLE-ABS(retest))) OR TITLE-ABS(stability) OR TITLE-ABS(interrater) OR TITLE-ABS(inter-rater) OR TITLE-ABS(intrarater) OR TITLE-ABS(intra-rater) OR TITLE-ABS(intertester) OR TITLE-ABS(inter-tester) OR TITLE-ABS(intratester) OR TITLE-ABS(intra-tester) OR TITLE-ABS(interobserver) OR TITLE-ABS(inter-observer) OR TITLE-ABS(intraobserver) OR TITLE-ABS(intra-observer) OR TITLE-ABS(intertechnician) OR TITLE-ABS(inter-technician) OR TITLE-ABS(intratechnician) OR TITLE-ABS(intra-technician) OR TITLE-ABS(interexaminer) OR TITLE-ABS(inter-examiner) OR TITLE-ABS(intraexaminer) OR TITLE-ABS(intra-examiner) OR TITLE-ABS(interassay) OR TITLE-ABS(inter-assay) OR TITLE-ABS(intraassay) OR TITLE-ABS(intra-assay) OR TITLE-ABS(interindividual) OR TITLE-ABS(inter-individual) OR TITLE-ABS(intraindividual) OR TITLE-ABS(intra-individual) OR TITLE-ABS(interparticipant) OR TITLE-ABS(inter-participant) OR TITLE-ABS(intraparticipant) OR TITLE-ABS(intra-participant) OR TITLE-ABS(kappa) OR TITLE-ABS(kappa's) OR TITLE-ABS(kappas) OR TITLE-ABS-KEY(repeatab*) OR ((TITLE-ABS-KEY(replicab*) OR TITLE-ABS-KEY(repeated)) AND (TITLE-ABS-KEY(measure) OR TITLE-ABS-KEY(measures) OR TITLE-ABS-KEY(findings) OR TITLE-ABS-KEY(result) OR TITLE-ABS-KEY(results) OR TITLE-ABS-KEY(test) OR TITLE-ABS-KEY(tests))) OR TITLE-ABS(generaliza*) OR TITLE-ABS(generalisa*) OR TITLE-ABS(concordance) OR (TITLE-ABS(intraclass) AND TITLE-ABS(correlation*)) OR TITLE-ABS(discriminative) OR TITLE-ABS("known group") OR TITLE-ABS("factor analysis") OR TITLE-ABS("factor analyses") OR TITLE-ABS("factor structure") OR TITLE-ABS("factor structures") OR TITLE-ABS(dimension*) OR TITLE-ABS(subscale*) OR (TITLE-ABS(multitrait) AND TITLE-ABS(scaling) AND (TITLE-ABS(analysis) OR TITLE-ABS(analyses))) OR TITLE-ABS("item discriminant") OR TITLE-ABS("interscale correlation*") OR TITLE-ABS(error) OR TITLE-ABS(errors) OR TITLE-ABS("individual variability") OR TITLE-ABS("interval variability") OR TITLE-ABS("rate variability") OR (TITLE-ABS(variability) AND (TITLE-ABS(analysis) OR TITLE-ABS(values))) OR (TITLE-ABS(uncertainty) AND (TITLE-ABS(measurement) OR TITLE-ABS(measuring))) OR TITLE-ABS("standard error of measurement") OR TITLE-ABS(sensitiv*) OR TITLE-ABS(responsive*) OR (TITLE-ABS(limit) AND TITLE-ABS(detection)) OR TITLE-ABS("minimal detectable concentration") OR TITLE-ABS(interpretab*) OR ((TITLE-ABS(minimal) OR TITLE-ABS(minimally) OR TITLE-ABS(clinical) OR TITLE-ABS(clinically)) AND (TITLE-ABS(important) OR TITLE-ABS(significant) OR TITLE-ABS(detectable)) AND (TITLE-ABS(change) OR TITLE-ABS(difference))) OR (TITLE-ABS(small*) AND (TITLE-ABS(real) OR TITLE-ABS(detectable)) AND (TITLE-ABS(change) OR TITLE-ABS(difference))) OR TITLE-ABS("meaningful change") OR TITLE-ABS("ceiling effect") OR TITLE-ABS("floor effect") OR TITLE-ABS("Item response model") OR TITLE-ABS(IRT) OR TITLE-ABS(Rasch) OR TITLE-ABS("Differential item functioning") OR TITLE-ABS(DIF) OR TITLE-ABS("computer adaptive testing") OR TITLE-ABS("item bank") OR TITLE-ABS("cross-cultural equivalence")))

Web of Science (142 records, 11 May)

(ALL="Angioedemas, Hereditary" OR ALL="Hereditary Angioedema" OR ALL="Hereditary Angioedemas" OR ALL="Angioedema, Hereditary" OR ALL="Hereditary angioneurotic edema" OR ALL="familial angioneurotic edema" OR ALL="Hereditary angioneurotic oedema") AND (ALL="Quality of Life" OR ALL="Life Quality" OR ALL="Health Related Quality Of Life" OR ALL="Health-Related Quality Of Life" OR ALL=HRQOL OR ALL=QoL) AND ((ALL="Instrumentation" OR ALL="Methods" OR ALL="Validation Studies" OR ALL="Comparative Study" OR ALL=psychometrics OR (TI=psychometr* OR AB=psychometr*) OR ALL=clinimetr* OR ALL=clinometr* OR ALL="outcome assessment, health care" OR (TI="outcome assessment" OR AB="outcome assessment") OR ALL="outcome measure*" OR ALL="observer variation" OR (TI="observer variation" OR AB="observer variation") OR ALL="Health Status Indicators" OR ALL="reproducibility of results" OR (TI=reproducib* OR AB=reproducib*) OR ALL="discriminant analysis" OR (TI=reliab* OR AB=reliab*) OR (TI=unreliab* OR AB=unreliab*) OR (TI=valid* OR AB=valid*) OR (TI="coefficient of variation" OR AB="coefficient of variation") OR (TI=coefficient OR AB=coefficient) OR (TI=homogeneity OR AB=homogeneity) OR (TI=homogeneous OR AB=homogeneous) OR (TI="internal consistency" OR AB="internal consistency") OR ((TI=cronbach* OR AB=cronbach*) AND ((TI=alpha OR AB=alpha) OR (TI=alphas OR AB=alphas))) OR ((TI=item OR AB=item) AND ((TI=correlation* OR AB=correlation*) OR (TI=selection* OR AB=selection*) OR (TI=reduction* OR AB=reduction*))) OR ALL=agreement OR ALL=precision OR ALL=imprecision OR ALL="precise values" OR (TI=test-retest OR AB=test-retest) OR ((TI=test OR AB=test) AND (TI=retest OR AB=retest)) OR ((TI=reliab* OR AB=reliab*) AND ((TI=test OR AB=test) OR (TI=retest OR AB=retest))) OR (TI=stability OR AB=stability) OR (TI=interrater OR AB=interrater) OR (TI=inter-rater OR AB=inter-rater) OR (TI=intrarater OR AB=intrarater) OR (TI=intra-rater OR AB=intra-rater) OR (TI=intertester OR AB=intertester) OR (TI=inter-tester OR AB=inter-tester) OR (TI=intratester OR AB=intratester) OR (TI=intra-tester OR AB=intra-tester) OR (TI=interobserver OR AB=interobserver) OR (TI=inter-observer OR AB=inter-observer) OR (TI=intraobserver OR AB=intraobserver) OR (TI=intra-observer OR AB=intra-observer) OR (TI=intertechnician OR AB=intertechnician) OR (TI=inter-technician OR AB=inter-technician) OR (TI=intratechnician OR AB=intratechnician) OR (TI=intra-technician OR AB=intra-technician) OR (TI=interexaminer OR AB=interexaminer) OR (TI=inter-examiner OR AB=inter-examiner) OR (TI=intraexaminer OR AB=intraexaminer) OR (TI=intra-examiner OR AB=intra-examiner) OR (TI=interassay OR AB=interassay) OR (TI=inter-assay OR AB=inter-assay) OR (TI=intraassay OR AB=intraassay) OR (TI=intra-assay OR AB=intra-assay) OR (TI=interindividual OR AB=interindividual) OR (TI=inter-individual OR AB=inter-individual) OR (TI=intraindividual OR AB=intraindividual) OR (TI=intra-individual OR AB=intra-individual) OR (TI=interparticipant OR AB=interparticipant) OR (TI=inter-participant OR AB=inter-participant) OR (TI=intraparticipant OR AB=intraparticipant) OR (TI=intra-participant OR AB=intra-participant) OR (TI=kappa OR AB=kappa) OR (TI=kappa's OR AB=kappa's) OR (TI=kappas OR AB=kappas) OR ALL=repeatab* OR ((ALL=replicab* OR ALL=repeated) AND (ALL=measure OR ALL=measures OR ALL=findings OR ALL=result OR ALL=results OR ALL=test OR ALL=tests)) OR (TI=generaliza* OR AB=generaliza*) OR (TI=generalisa* OR AB=generalisa*) OR (TI=concordance OR AB=concordance) OR ((TI=intraclass OR AB=intraclass) AND (TI=correlation* OR AB=correlation*)) OR (TI=discriminative OR AB=discriminative) OR (TI="known group" OR AB="known group") OR (TI="factor analysis" OR AB="factor analysis") OR (TI="factor analyses" OR AB="factor analyses") OR (TI="factor structure" OR AB="factor structure") OR (TI="factor structures" OR AB="factor structures") OR (TI=dimension* OR AB=dimension*) OR (TI=subscale* OR AB=subscale*) OR ((TI=multitrait OR AB=multitrait) AND (TI=scaling OR AB=scaling) AND ((TI=analysis OR AB=analysis) OR (TI=analyses OR AB=analyses))) OR (TI="item discriminant" OR AB="item discriminant") OR (TI="interscale correlation*" OR AB="interscale correlation*") OR (TI=error OR AB=error) OR (TI=errors OR AB=errors) OR (TI="individual variability" OR AB="individual variability") OR (TI="interval variability" OR AB="interval variability") OR (TI="rate variability" OR AB="rate variability") OR ((TI=variability OR AB=variability) AND ((TI=analysis OR AB=analysis) OR (TI=values OR AB=values))) OR ((TI=uncertainty OR AB=uncertainty) AND ((TI=measurement OR AB=measurement) OR (TI=measuring OR AB=measuring))) OR (TI="standard error of measurement" OR AB="standard error of measurement") OR (TI=sensitiv* OR AB=sensitiv*) OR (TI=responsive* OR AB=responsive*) OR ((TI=limit OR AB=limit) AND (TI=detection OR AB=detection)) OR (TI="minimal detectable concentration" OR AB="minimal detectable concentration") OR (TI=interpretab* OR AB=interpretab*) OR (((TI=minimal OR AB=minimal) OR (TI=minimally OR AB=minimally) OR (TI=clinical OR AB=clinical) OR (TI=clinically OR AB=clinically)) AND ((TI=important OR AB=important) OR (TI=significant OR AB=significant) OR (TI=detectable OR AB=detectable)) AND ((TI=change OR AB=change) OR (TI=difference OR

AB=difference))) OR ((TI=small* OR AB=small*) AND ((TI=real OR AB=real) OR (TI=detectable OR AB=detectable)) AND ((TI=change OR AB=change) OR (TI=difference OR AB=difference))) OR (TI="meaningful change" OR AB="meaningful change") OR (TI="ceiling effect" OR AB="ceiling effect") OR (TI="floor effect" OR AB="floor effect") OR (TI="Item response model" OR AB="Item response model") OR (TI=IRT OR AB=IRT) OR (TI=Rasch OR AB=Rasch) OR (TI="Differential item functioning" OR AB="Differential item functioning") OR (TI=DIF OR AB=DIF) OR (TI="computer adaptive testing" OR AB="computer adaptive testing") OR (TI="item bank" OR AB="item bank") OR (TI="cross-cultural equivalence" OR AB="cross-cultural equivalence")))

Embase (695 records, 11 May)

('angioedemas, hereditary'/exp OR 'hereditary angioedema' OR 'hereditary angioedemas' OR 'angioedema, hereditary' OR 'hereditary angioneurotic edema' OR 'familial angioneurotic edema' OR 'hereditary angioneurotic oedema') AND ('quality of life'/exp OR 'life quality' OR 'health related quality of life' OR 'health-related quality of life' OR hrqol OR qol) AND ('instrumentation' OR 'methods' OR term:it OR 'psychometrics'/exp OR psychometr*:ti,ab OR clinimetr* OR clinometr* OR 'outcome assessment (health care)'/exp OR 'outcome assessment':ti,ab OR 'outcome measure*' OR 'observer variation'/exp OR 'observer variation':ti,ab OR 'health status indicators'/exp OR 'reproducibility of results'/exp OR reproducib*:ti,ab OR 'discriminant analysis'/exp OR reliab*:ti,ab OR unreliab*:ti,ab OR valid*:ti,ab OR 'coefficient of variation':ti,ab OR coefficient:ti,ab OR homogeneity:ti,ab OR homogeneous:ti,ab OR 'internal consistency':ti,ab OR (cronbach*:ti,ab AND (alpha:ti,ab OR alphas:ti,ab)) OR (item:ti,ab AND (correlation*:ti,ab OR selection*:ti,ab OR reduction*:ti,ab)) OR agreement OR precision OR imprecision OR 'precise values' OR 'test retest':ti,ab OR (test:ti,ab AND retest:ti,ab) OR (reliab*:ti,ab AND (test:ti,ab OR retest:ti,ab)) OR stability:ti,ab OR interrater:ti,ab OR 'inter rater':ti,ab OR intrarater:ti,ab OR 'intra rater':ti,ab OR intertester:ti,ab OR 'inter tester':ti,ab OR intratester:ti,ab OR 'intra tester':ti,ab OR interobserver:ti,ab OR 'inter observer':ti,ab OR intraobserver:ti,ab OR 'intra observer':ti,ab OR intertechnician:ti,ab OR 'inter technician':ti,ab OR intratechnician:ti,ab OR 'intra technician':ti,ab OR interexaminer:ti,ab OR 'inter examiner':ti,ab OR intraexaminer:ti,ab OR 'intra examiner':ti,ab OR interassay:ti,ab OR 'inter assay':ti,ab OR intraassay:ti,ab OR 'intra assay':ti,ab OR interindividual:ti,ab OR 'inter individual':ti,ab OR intraindividual:ti,ab OR 'intra individual':ti,ab OR interparticipant:ti,ab OR 'inter participant':ti,ab OR intraparticipant:ti,ab OR 'intra participant':ti,ab OR kappa:ti,ab OR repeatab* OR ((replicab* OR repeated) AND (measure OR measures OR findings OR result OR results OR test OR tests)) OR generaliza*:ti,ab OR generalisa*:ti,ab OR concordance:ti,ab OR (intraclass:ti,ab AND correlation*:ti,ab) OR discriminative:ti,ab OR 'known group':ti,ab OR 'factor analysis':ti,ab OR 'factor analyses':ti,ab OR 'factor structure':ti,ab OR 'factor structures':ti,ab OR dimension*:ti,ab OR subscale*:ti,ab OR (multitrait:ti,ab AND scaling:ti,ab AND (analysis:ti,ab OR analyses:ti,ab)) OR 'item discriminant':ti,ab OR 'interscale correlation*':ti,ab OR error:ti,ab OR errors:ti,ab OR 'individual variability':ti,ab OR 'interval variability':ti,ab OR 'rate variability':ti,ab OR (variability:ti,ab AND (analysis:ti,ab OR values:ti,ab)) OR (uncertainty:ti,ab AND (measurement:ti,ab OR measuring:ti,ab)) OR 'standard error of measurement':ti,ab OR sensitiv*:ti,ab OR responsive*:ti,ab OR (limit:ti,ab AND detection:ti,ab) OR 'minimal detectable concentration':ti,ab OR interpretab*:ti,ab OR ((minimal:ti,ab OR minimally:ti,ab OR clinical:ti,ab OR clinically:ti,ab) AND (important:ti,ab OR significant:ti,ab OR detectable:ti,ab) AND (change:ti,ab OR difference:ti,ab)) OR (small*:ti,ab AND (real:ti,ab OR detectable:ti,ab) AND (change:ti,ab OR difference:ti,ab)) OR 'meaningful change':ti,ab OR 'ceiling effect':ti,ab OR 'floor effect':ti,ab OR 'item response model':ti,ab OR irt:ti,ab OR rasch:ti,ab OR 'differential item functioning':ti,ab OR dif:ti,ab OR 'computer adaptive testing':ti,ab OR 'item bank':ti,ab OR 'cross-cultural equivalence':ti,ab)

CINAHL (14 records)

((MH "Angioedemas, Hereditary"+) OR "Hereditary Angioedema" OR "Hereditary Angioedemas" OR "Angioedema, Hereditary" OR "Hereditary angioneurotic edema" OR "familial angioneurotic edema" OR "Hereditary angioneurotic oedema") AND ((MH "Quality of Life"+) OR "Life

Quality" OR "Health Related Quality Of Life" OR "Health-Related Quality Of Life" OR HRQOL OR QoL) AND (("Instrumentation" OR "Methods" OR (PT "Validation Studies") OR (PT "Comparative Study") OR (MH psychometrics+) OR (TI psychometr* OR AB psychometr*) OR clinimetr* OR clinometr* OR (MH "outcome assessment (health care)+") OR (TI "outcome assessment" OR AB "outcome assessment") OR "outcome measure*" OR (MH "observer variation+") OR (TI "observer variation" OR AB "observer variation") OR (MH "Health Status Indicators+") OR (MH "reproducibility of results+") OR (TI reproducib* OR AB reproducib*) OR (MH "discriminant analysis+") OR (TI reliab* OR AB reliab*) OR (TI unreliab* OR AB unreliab*) OR (TI valid* OR AB valid*) OR (TI "coefficient of variation" OR AB "coefficient of variation") OR (TI coefficient OR AB coefficient) OR (TI homogeneity OR AB homogeneity) OR (TI homogeneous OR AB homogeneous) OR (TI "internal consistency" OR AB "internal consistency") OR ((TI cronbach* OR AB cronbach*) AND ((TI alpha OR AB alpha) OR (TI alphas OR AB alphas))) OR ((TI item OR AB item) AND ((TI correlation* OR AB correlation*) OR (TI selection* OR AB selection*) OR (TI reduction* OR AB reduction*))) OR agreement OR precision OR imprecision OR "precise values" OR (TI test-retest OR AB test-retest) OR ((TI test OR AB test) AND (TI retest OR AB retest)) OR ((TI reliab* OR AB reliab*) AND ((TI test OR AB test) OR (TI retest OR AB retest))) OR (TI stability OR AB stability) OR (TI interrater OR AB interrater) OR (TI inter-rater OR AB inter-rater) OR (TI intrarater OR AB intrarater) OR (TI intra-rater OR AB intra-rater) OR (TI intertester OR AB intertester) OR (TI inter-tester OR AB inter-tester) OR (TI intratester OR AB intratester) OR (TI intra-tester OR AB intra-tester) OR (TI interobserver OR AB interobserver) OR (TI inter-observer OR AB inter-observer) OR (TI intraobserver OR AB intraobserver) OR (TI intra-observer OR AB intra-observer) OR (TI intertechnician OR AB intertechnician) OR (TI inter-technician OR AB inter-technician) OR (TI intratechnician OR AB intratechnician) OR (TI intra-technician OR AB intra-technician) OR (TI interexaminer OR AB interexaminer) OR (TI inter-examiner OR AB inter-examiner) OR (TI intraexaminer OR AB intraexaminer) OR (TI intra-examiner OR AB intra-examiner) OR (TI interassay OR AB interassay) OR (TI inter-assay OR AB inter-assay) OR (TI intraassay OR AB intraassay) OR (TI intra-assay OR AB intra-assay) OR (TI interindividual OR AB interindividual) OR (TI inter-individual OR AB inter-individual) OR (TI intraindividual OR AB intraindividual) OR (TI intra-individual OR AB intra-individual) OR (TI interparticipant OR AB interparticipant) OR (TI inter-participant OR AB inter-participant) OR (TI intraparticipant OR AB intraparticipant) OR (TI intra-participant OR AB intra-participant) OR (TI kappa OR AB kappa) OR (TI kappa’s OR AB kappa’s) OR (TI kappas OR AB kappas) OR repeatab* OR ((replicab* OR repeated) AND (measure OR measures OR findings OR result OR results OR test OR tests)) OR (TI generaliza* OR AB generaliza*) OR (TI generalisa* OR AB generalisa*) OR (TI concordance OR AB concordance) OR ((TI intraclass OR AB intraclass) AND (TI correlation* OR AB correlation*)) OR (TI discriminative OR AB discriminative) OR (TI "known group" OR AB "known group") OR (TI "factor analysis" OR AB "factor analysis") OR (TI "factor analyses" OR AB "factor analyses") OR (TI "factor structure" OR AB "factor structure") OR (TI "factor structures" OR AB "factor structures") OR (TI dimension* OR AB dimension*) OR (TI subscale* OR AB subscale*) OR ((TI multitrait OR AB multitrait) AND (TI scaling OR AB scaling) AND ((TI analysis OR AB analysis) OR (TI analyses OR AB analyses))) OR (TI "item discriminant" OR AB "item discriminant") OR (TI "interscale correlation*" OR AB "interscale correlation*") OR (TI error OR AB error) OR (TI errors OR AB errors) OR (TI "individual variability" OR AB "individual variability") OR (TI "interval variability" OR AB "interval variability") OR (TI "rate variability" OR AB "rate variability") OR ((TI variability OR AB variability) AND ((TI analysis OR AB analysis) OR (TI values OR AB values))) OR ((TI uncertainty OR AB uncertainty) AND ((TI measurement OR AB measurement) OR (TI measuring OR AB measuring))) OR (TI "standard error of measurement" OR AB "standard error of measurement") OR (TI sensitiv* OR AB sensitiv*) OR (TI responsive* OR AB responsive*) OR ((TI limit OR AB limit) AND (TI detection OR AB detection)) OR (TI "minimal detectable concentration" OR AB "minimal detectable concentration") OR (TI interpretab* OR AB interpretab*) OR (((TI minimal OR AB minimal) OR (TI minimally OR AB minimally) OR (TI clinical OR AB clinical) OR (TI clinically OR AB clinically)) AND ((TI important OR AB important) OR (TI significant OR AB significant) OR (TI detectable OR AB detectable)) AND ((TI change OR AB change) OR (TI difference OR AB difference))) OR ((TI small* OR AB small*) AND ((TI real OR AB real) OR (TI detectable OR AB detectable)) AND ((TI change OR AB change) OR (TI difference OR AB difference))) OR (TI "meaningful change" OR AB "meaningful change") OR (TI "ceiling effect" OR AB "ceiling effect") OR (TI "floor effect" OR AB "floor effect") OR (TI "Item response model" OR AB "Item response model") OR (TI IRT OR AB IRT) OR (TI Rasch OR AB Rasch) OR (TI "Differential item functioning" OR AB "Differential item functioning") OR (TI DIF OR AB DIF) OR (TI "computer adaptive

testing" OR AB "computer adaptive testing") OR (TI "item bank" OR AB "item bank") OR (TI "cross-cultural equivalence" OR AB "cross-cultural equivalence")))
