# Supplementary material for: A COSMIN systematic review of instruments for evaluating health-related quality of life in people with Hereditary Angioedema
Source: Health Qual Life Outcomes. 2025 Feb 13;23:12. doi: 10.1186/s12955-025-02342-6 (PMC11823193; doi:10.1186/s12955-025-02342-6)
Supplement: Supplementary file 4 — Supplementary Material 4. [file 12955_2025_2342_MOESM4_ESM.docx]

# Supplementary File 4. Characteristics of the included study populations

|  |  | **Population** | | | **Disease characteristics** | **Instrument administration** | | |
| --- | --- | --- | --- | --- | --- | --- | --- | --- |
| **PROM** | **First author (year)** | **N** | **Age**  **Mean (SD, range) yr** | **Gender**  **% female** | **Disease/**  **Type** | **Setting (timing)** | **Country** | **Language** |
| SF-36 | Gomide et.al  (2013) | 35 | Mean 40.7(±16.6) yrs  Range ≥15 yrs | 71.4% | HAE C1- INH (type I or II) | **NR** | Brazil | Portuguese |
| SF-36v2 | Jindal et. al  (2017) | 21 | Mean 42.3(±13.7) yrs Range ≥18 yrs | 95.2% | HAE C1- INH (type I or II) | Canadian HAE patient network | Canada | English  French |
|  | Palao‑Ocharan et. all (2022) | 290 (phase 1)  20 (phase 2) | Mean 41.5 (±14.5) yrs  Range ≥18 yrs | 69.0% | HAE C1- INH (type I or II) | Hospital or home | Argentina,  Austria,  Brazil,  Canada,  Denmark,  Germany,  Hungary,  Israel,  Poland,  Romania,  Spain | Native languages. |
| HAE-QoL | Prior et al.  (2012) | 45 patients  8 experts | Mean 39 (18–74) yrs  Range ≥18 yrs | 64.4% | HAE-C1-INH (type I or II) | National multi-centre | Spain | Spanish |
|  | Prior et al.  (2016) | 290 patients  15 experts | Mean 41.5 (±14.6) yrs  Range ≥18 yrs | 69.0% | HAE C1- INH (type I or II) | Health clinic and follow up mailing | Argentina,  Austria,  Brazil,  Canada,  Denmark,  Germany,  Hungary,  Israel,  Poland,  Romania,  Spain,  Italy,  Macedonia,  United Kingdom,  Panama,  China,  France. | American English and Native languages. |
| HAEA-QoL | Busse et al.  (2019) | 168 | Range <18 yrs = 7.1 %  Range 18-30 yrs: 13.7 %  Range 31-40 yrs: 16.7 %  Range 41-50 yrs: 20.2 %  Range 51-60 yrs: 25.6 %  Range 61-70 yrs: 13.1 %  Range 71-80 yrs: 3.6 % | 73.2% | HAE C1- INH (type I or II) or not know type | Survey and focus group on HAE Association [HAEA] Patient  Summit 2015 | United States | American English |
| AE- QoL | Vanya et al.  (2023) | 40 patients and 7 experts (first phase) 64 patients (second phase) | Mean 41(±20.89) yrs;  Range 13-72 yrs | 69.0% | HAE C1- INH (type I or II) | International telephone interviews, and cross-sectional data from phase 3 trial. | Canada, France, Spain, Germany, United Kingdom, United States | Native languages |

SF-36v2: Short Form 36-item Health Survey Version 2.0; HAE-QoL: Hereditary angioedema quality of life; SF-36: Short Form 36-item Health Survey; HAEA-

QoL: Hereditary Angioedema Association quality of life; AE-QoL: Angioedema Quality of Life Questionnaire; NR: Not Reported
