# Supplementary material for: A COSMIN systematic review of instruments for evaluating health-related quality of life in people with Hereditary Angioedema
Source: Health Qual Life Outcomes. 2025 Feb 13;23:12. doi: 10.1186/s12955-025-02342-6 (PMC11823193; doi:10.1186/s12955-025-02342-6)
Supplement: Supplementary file 5 — Supplementary Material 5. [file 12955_2025_2342_MOESM5_ESM.docx]

**Supplementary File 5**

**Definitions of quality levels**

| **Quality level** | **Definition** |
| --- | --- |
| High | We are very confident that the true measurement property lies close to that of the estimate of the measurement property |
| Moderate | We are moderately confident in the measurement property estimate: the true measurement property is likely to be close to the estimate of the measurement property, but there is a possibility that it is substantially different |
| Low | Our confidence in the measurement property estimate is limited: the true measurement property may be substantially different from the estimate of the measurement property |
| Very low | We have very little confidence in the measurement property estimate: the true measurement property is likely to be substantially different from the estimate of the measurement property |

These definitions were adapted from the GRADE approach. Information on how to downgrade is described in detail in the COSMIN user manual.

**Modified GRADE approach for grading the quality of evidence**

| **Quality of evidence** | **Lower if** |
| --- | --- |
| High | Risk of bias  −1 Serious  −2 Very serious  −3 Extremely serious Inconsistency  −1 Serious  −2 Very serious Imprecision  −1 total *n* = 50–100  −2 total *n* < 50 Indirectness  −1 Serious  −2 Very serious |
| Moderate |  |
| Low |  |
| Very low |  |

The starting point is the assumption that the evidence is of high quality. The quality of evidence is subsequently downgraded with one or two levels for each factor (i.e., risk of bias, inconsistency, imprecision, indirectness) to moderate, low, or very low when there is risk of bias (low study quality), (unexplained) inconsistency in results, or indirect results. Information on how to downgrade is described in detail in the COSMIN user manual.

*n* = sample size
