# Supplementary material for: A COSMIN systematic review of instruments for evaluating health-related quality of life in people with Hereditary Angioedema
Source: Health Qual Life Outcomes. 2025 Feb 13;23:12. doi: 10.1186/s12955-025-02342-6 (PMC11823193; doi:10.1186/s12955-025-02342-6)
Supplement: Supplementary file 6 — Supplementary Material 6. [file 12955_2025_2342_MOESM6_ESM.docx]

**Supplementary File 6. COSMIN Risk of Bias checklist**

| **Boxes of the COSMIN Risk of Bias checklist** |
| --- |
| *Content validity* |
| Box 1. PROM development |
| Box 2. Content validity |
| *Internal structure* |
| Box 3. Structural validity |
| Box 4. Internal consistency |
| Box 5. Cross-cultural validity\measurement invariance |
| *Remaining measurement properties* |
| Box 6. Reliability |
| Box 7. Measurement error |
| Box 8. Criterion validity |
| Box 9. Hypotheses testing for construct validity |
| Box 10. Responsiveness |
